# Rapid Discovery and Functional Characterization of Terpene Synthases from Four Endophytic Xylariaceae

**DOI:** 10.1371/journal.pone.0146983

**Published:** 2016-02-17

**Authors:** Weihua Wu, William Tran, Craig A. Taatjes, Jorge Alonso-Gutierrez, Taek Soon Lee, John M. Gladden

**Affiliations:** 1 Biomass Science & Conversion Technologies, Sandia National Laboratories, Livermore, California, United States of America; 2 Combustion Chemistry Department, Sandia National Laboratories, Livermore, California, United States of America; 3 Physical Biosciences Division, Lawrence Berkeley National Laboratory, Berkeley, California, United States of America; 4 Joint BioEnergy Institute, Emeryville, California, United States of America; Michigan State University, UNITED STATES

## Abstract

Endophytic fungi are ubiquitous plant endosymbionts that establish complex and poorly understood relationships with their host organisms. Many endophytic fungi are known to produce a wide spectrum of volatile organic compounds (VOCs) with potential energy applications, which have been described as "mycodiesel". Many of these mycodiesel hydrocarbons are terpenes, a chemically diverse class of compounds produced by many plants, fungi, and bacteria. Due to their high energy densities, terpenes, such as pinene and bisabolene, are actively being investigated as potential "drop-in" biofuels for replacing diesel and aviation fuel. In this study, we rapidly discovered and characterized 26 terpene synthases (TPSs) derived from four endophytic fungi known to produce mycodiesel hydrocarbons. The TPS genes were expressed in an *E*. *coli* strain harboring a heterologous mevalonate pathway designed to enhance terpene production, and their product profiles were determined using Solid Phase Micro-Extraction (SPME) and GC-MS. Out of the 26 TPS’s profiled, 12 TPS’s were functional, with the majority of them exhibiting both monoterpene and sesquiterpene synthase activity.

## Introduction

Endophytic fungi have evolved to live within plant tissues without causing overt harm to their hosts. This endosymbiotic relationship involves continual interactions between host and fungi using a variety of signals, including exchange of secondary metabolites, that elicit specific biological responses [1]. Recent studies aimed at characterizing the various secondary metabolites produced by endophytic fungi revealed that many of these fungi emit a wide spectrum of volatile organic compounds (VOCs) while growing on plant and agricultural residues [2–9]. Not only do these VOCs play important roles in the biology of these fungi, they also supply a rich reservoir of potential compounds for medicinal and industrial applications. Many of these VOCs are hydrocarbons and other oxygenated compounds that have been referred to as “mycodiesel” due to their high energy density and near zero oxygen content, which make them compatible with the existing engines and great “drop-in” biofuel candidates. A large fraction of “mycodiesel” compounds are terpenes and their derivatives. Terpenes, or isoprenoids, are one of the most diverse class of natural products with more than 55,000 different terpenoids known [10]. They have a myriad of biological functions (antibiotics, hormones, anticancer agents, etc.) and industrial applications (flavorings, fragrances, and biofuels, etc.)[11–17]. For example, mono- and sesquiterpenes are the major components of VOCs produced by the endophytes *Hypoxylon sp*. CI4A, *Hypoxylon sp*. CO27, *Hypoxylon sp*. EC38, and *Daldinia eschscholzii* EC12 when grown on potato dextrose. These organisms also produce lower levels of other non-terpene compounds, such as ketones (11% in CI4A culture) and alcohols (20% in EC12 culture) that have potential biofuel applications, indicating that there is a myriad of potential useful biosynthetic pathways present in these organisms. To make use of these compounds in industry, the biosynthetic pathways that generate them need to be elucidated, enabling them to either be manipulated in their native host to increase productivity, or to be ported into an existing industrial host where their production can be more easily controlled [9].

**Fig 1:**
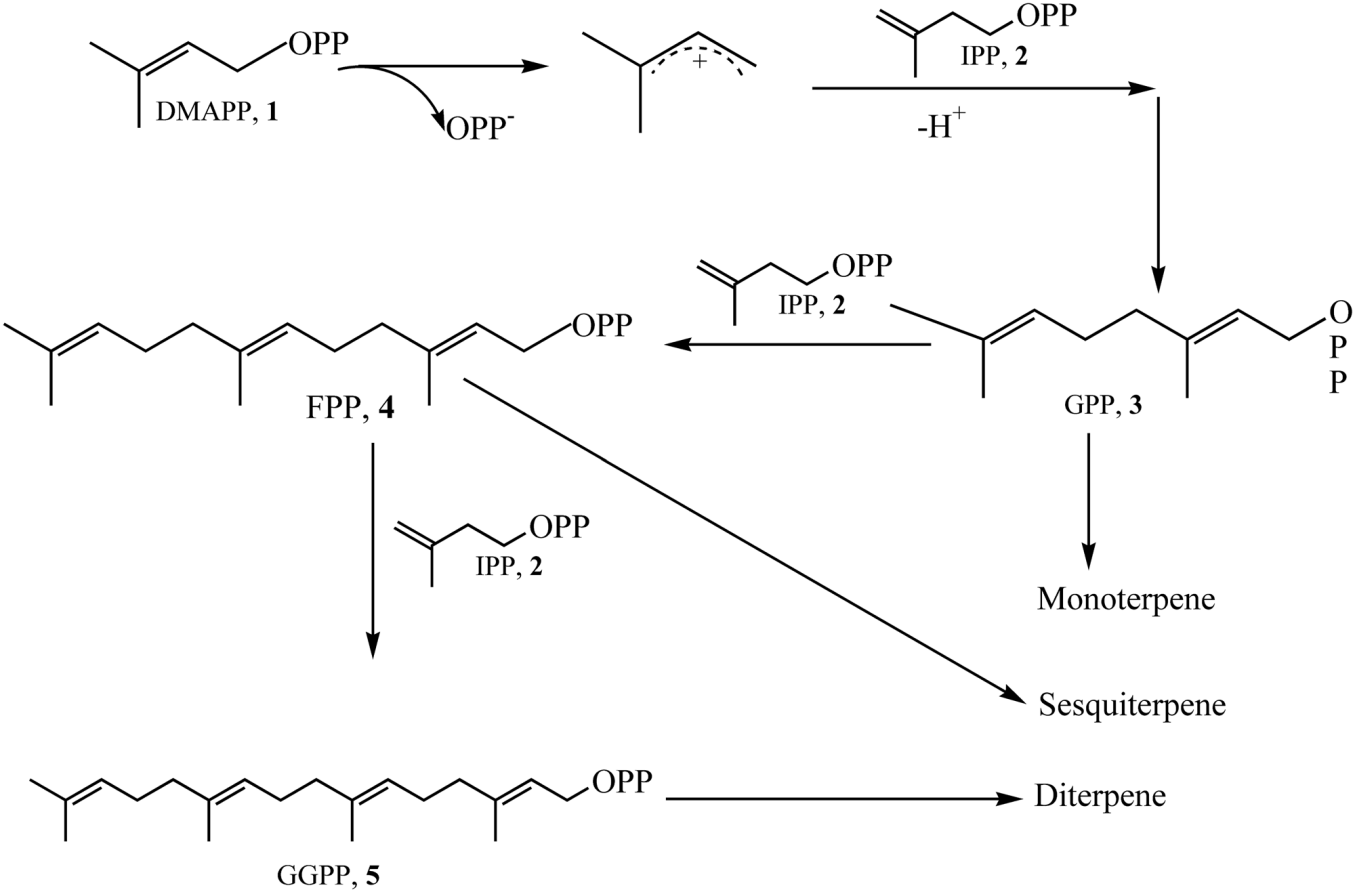
Mechanism for the biosynthesis of GPP, FPP, GGPP and corresponding terpene compounds [1].

The biosynthesis of isoprenoids is well understood. Their universal building blocks are the C_5_ precursors isopentenyl pyrophosphate (IPP) and dimethylallyl pyrophosphate (DMAPP). Successive condensations of DMAPP with one or more IPP in a 1–4 fashion gives rise to linear isoprenyl diphosphate compounds of various chain lengths: geranyl pyrophosphate (C_10_, GPP), farnesyl pyrophosphate (C_15_, FPP), and geranylgeranyl pyrophosphate (C_20_,GGPP; Fig 1). These precursors are then catalyzed by terpene synthases (TPSs) into monoterpenes (C_10_), sesquiterpenes (C_15_), diterpenes (C_20_), and other compounds. Most terpene synthases belong to either the terpene synthase type I or type II superfamily, which can be distinguished by distinct motifs [1, 18]. The catalytic reaction of type I terpene synthase involves carbocation formation by abstraction of two diphosphate groups from the substrate through complexation to two highly conserved motifs: the aspartate rich motif (DDXXD) and the NSE/DTE triad ND (L/I/V)XSXXXE. The type II terpene synthase superfamily have a highly conserved DXDD motif that facilitates the formation of a carbocation by protonation of an epoxide or olefin [1]. To date, genome sequencing has uncovered more than a thousand different genes encoding terpene synthases in bacteria [19, 20], fungi [21, 22], and plants [23–25]. Recently, endophytic fungi have also been reported to produce a diverse spectrum of terpenes, including monoterpenes, sesquiterpenes, diterpenes, and other derivatives [2, 5, 7, 26, 27]. These terpenes are not only biologically active secondary metabolites with great pharmaceutical potential but they also have a high energy density, making them attractive renewable fossil fuel alternatives [2, 5, 28–30]. However, there are few reports describing the discovery and characterization of the terpene synthase genes that produce these compounds [31].

In this study, we undertook a systematical approach combining genome dataset mining, terpene biosynthetic pathway construction in *E*. *coli*, Solid Phase MicroExtraction (SPME), and GC-MS analysis to rapidly discover and characterize endophytic terpene synthases. We sequenced four endophytic fungi in the order of Xylariales (*Hypoxylon sp*. CI4A, *Hypoxylon sp*. CO27, *Hypoxylon sp*. EC38, and *Daldinia eschscholzii* EC12) and mined their genomes for potential TPS genes. A total of 26 putative TPS genes were identified, of which 12 were functionally expressed in *E*. *coli* and produced a wide array of monoterpenes and sesquiterpenes.

## Material and Methods

### The discovery and phylogenetic tree analysis of the putative endophytic terpene synthases

The putative endophyte TPS genes were identified by searching the endophyte genomes for terpene synthase Pfam functional domains. The protein sequences of the putative TPSs were downloaded from the endophyte genomes published by the Joint Genome Institute [32]. Secretion signal peptides were predicted using the Signal P4.1 online tool [33]. The endophytic TPS protein sequences were compared to each other and to several TPS from plants and other fungi. All protein sequences were aligned by Clustal W in MEGA 6.0 [34]. Neighbor joining trees were made by MEGA6.0 using the bootstrap method and Poisson model, with bar = 0.2 substitutions per amino acid residue. The sequence comparison of the endophytic TPSs with other plant and fungal TPSs was presented as rectangular trees, while the comparison amongst the endophytic TPSs was presented as a radiation tree.

### Stains and plasmids

*E*.*coli* strains DH10B and DH1 were used for cloning and production, respectively. Plasmids pJBEI-3122, pBbE1a, and pBbE2k were previously reported [35]. Plasmid pJBEI-3122 contains genes encoding seven enzymes (AtoB, HMGS, HMGR, MK, PMK, PMD, and IDI) of the mevalonate pathway. The protein sequences of the TPSs in this study and the geranyl pyrophosphate synthase (GPPS, GenBank: AF513112.1, GPPS_Ag_) from *Abies grandis* (with the chloroplast signal peptide truncated) were used to generate codon optimized genes for expression in *E*.*coli*. A Ribosome Binding Site (RBS) for each putative terpene gene was created and optimized using an online RBS calculator [36]. All the DNA sequences containing the RBS site and TPS or GPPS gene, flanked by *BamH*I and *EcoR*I sites, were synthesized by Genscript.

### Reconstruction of the terpene biosynthetic pathway in *E*.*coli* strain DH1

Each synthesized TPS ORF, including the optimized RBS, was digested by the restriction enzymes *BamH*I and *EcoR*I and ligated by T4 DNA ligase (New England Biolabs, CA) into plasmid pBbE1a to create vector pBbE1a-TPS. The synthesized GPPS_Ag_ DNA fragment was digested by *BamH*I and *EcoR*I, and ligated into vector pBbE2k using T4 DNA ligase to generate the plasmid pBbE2k-GPPS_Ag_. The complete terpene biosynthetic pathway was reconstructed in *E*.*coli* strain DH1 by co-transforming all three plasmids pJBEI-3122, pBbE1a-TPS, and pBbE2k-GPPS_Ag_. The plasmids pJBEI-3122 and pBbE2k-GPPS_Ag_ were also co-transformed into strain DH1 as a negative control.

### Production of the terpene compounds in *E*.*coli*

The transformants containing each TPS gene were cultured in 15 mL of LB medium with 100 μg/L of ampicillin, 34 μg/L chloramphenicol, and 25 μg/L of kanamycin. The cultures were incubated at 37°C shaking at 220 rpm overnight. One mL of overnight culture was then inoculated into 20 mL of fresh EZ-rich medium (Teknova, CA) containing 20g/L glucose as well as the three aforementioned antibiotics and incubated at 37°C with shaking at 220 rpm until an OD_600nm_ of 0.8 was reached. Then terpene production was induced by adding isopropyl-β-D-1-thiogalactopyranoside (IPTG) at the final concentration of 1mM and incubating for another 20 hours at 30°C with shaking at 180 rpm. Terpenes were extracted after 48 hours.

### GC/MS analysis of terpene

The volatile terpene compounds in the headspace of each culture were analyzed by extracting VOCs with a preconditioned Solid-Phase Micro-Extraction (SPME) syringe consisting of 50/30 divinylbenzene/carboxen on polydimethylsiloxane on a Stable Flex fiber followed by GC-MS. The SPME fiber was explored into the headspace of each culture flask for an hour to saturate with the volatile terpene compounds produced by the various TPS-expressing strains. The syringe was then inserted into the injection port of a Varian 3800 gas chromatograph containing a 30mx0.25mm i.d DB waxer capillary column with a film thickness 0.25μm. The column temperature was programmed as follows: 60°C for 4 min, increasing to 120°C at 10°C/min and holding for 5 min, then increasing to 220°C at 20°C/min and holding for 2 min, then increasing to 250°C at 50°C/min and holding for 4 min. The carrier gas was ultra-high purity helium at a constant flow rate of 1 mL/min, and the initial column head pressure was 50Kpa. A two minute injection time was used to desorb the terpene compounds from the sampling fiber into an injection port (splitless mode, injection temperature—220°C) of the chromatograph coupled with a Saturn 2000 ion trap mass spectrometer. The MSD parameters were EI at 70eV, mass range was 30–500 Da, and the scan speed was 2 scans/sec.

GC-MS data deconvolution was performed using the Automated Mass Spectral Deconvolution and Identification System (AMDIS) spectral deconvolution software package (v. 2.70, NIST Gaithersburg). AMDIS deconvolution settings were as follows: resolution (medium), sensitivity (low), shape requirement (medium), and component width at 10. Spectral components were searched against the NIST 2011 mass spectral library, and only components with mass spectra match factors > 85% were reported as tentatively identified compounds. Compounds with peak areas >1% of the total peak area in the chromatogram are reported. A large number of terpenes were identified by GC-MS, and to confirm their identity, several commercially available terpene standards were purchased from Sigma-Aldrich and analyzed using the same methodology. They are listed in S7 Table. All terpenes mentioned in this manuscript that do not appear in in S7 Table are considered only to be putatively identified.

## Results and Discussion

### Identification of terpene synthase genes in four endophytic fungal genomes

**Fig 2:**
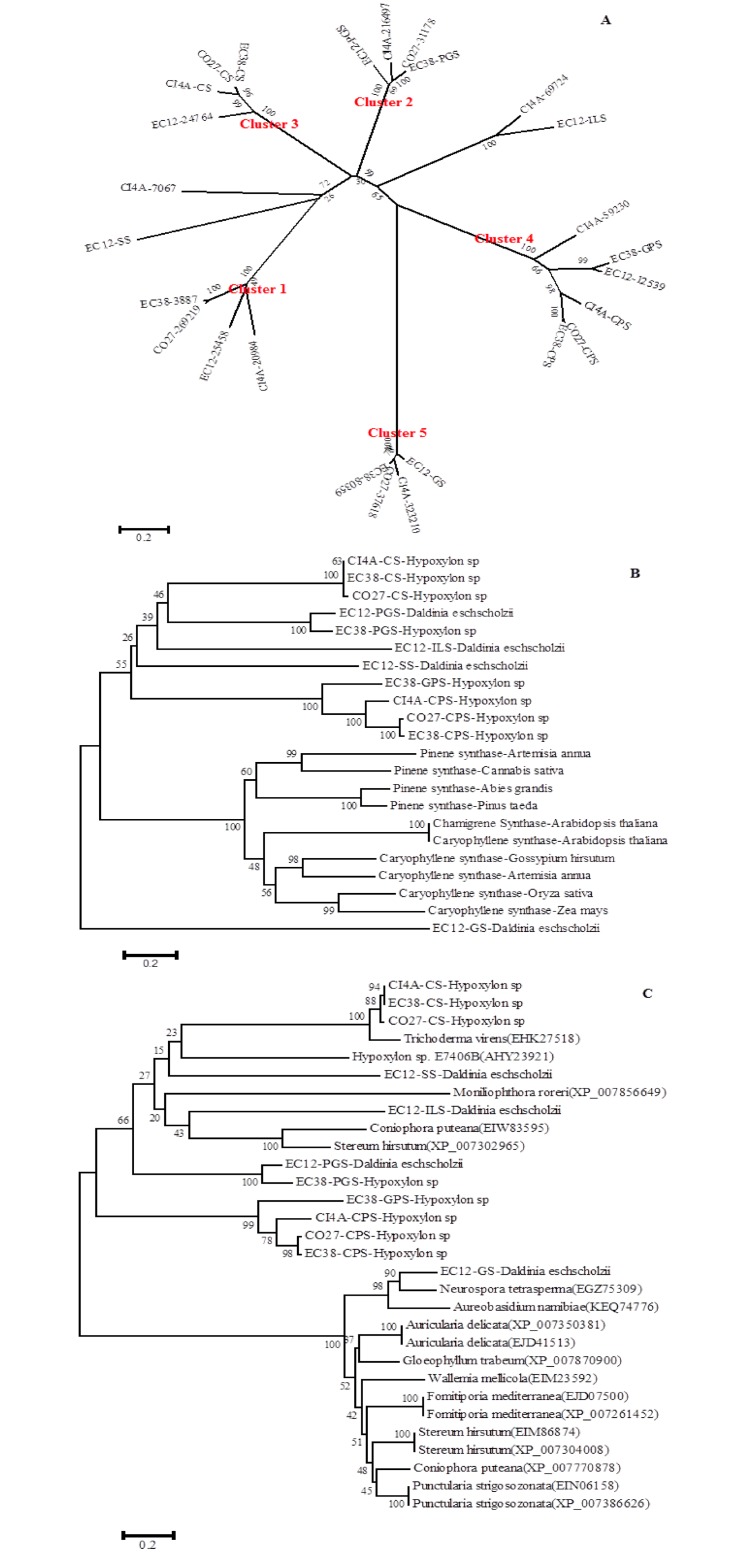
Phylogenetic tree analysis of Endophyte Terpene Synthases. A) Comparison of TPSs from four endophytic fungi in the genus Hypoxylon or Daldinia. A total of 26 TPSs from these fungi were grouped into five distinct clusters., B) Comparison of endophyte and plant TPSs., C) Comparison of endophyte and other fungal TPSs.

TPS genes were identified in the genomes of *Hypoxylon sp*. CI4A, *Hypoxylon sp*. CO27, *Hypoxylon sp*. EC38, and *Daldinia eschscholzii* EC12 by homology searches against conserved TPS domains. A total of 26 putative TPSs were identified in the genomes of these four endophytes, including 7 TPSs from CI4A, 5 from CO27, 6 from EC38, and 6 from EC12. Analysis of the protein sequences determined that none of these TPS harbor signal peptides. Protein sequence alignments with known TPSs determined that all the putative fungal TPSs fall into the type I terpene synthase superfamily and harbor a highly conserved aspartate-rich motif (DDXXD/E). Also, all but cluster 5 TPSs (Fig 2) have a (N/D)DXX(S/T)XX(K/R) (D/E) NSE/DTE triad consensus sequence, which possess a X(D/K)XXXSXXRE triad (Table 1). Phylogenetic analysis of the 26 putative TPSs grouped all but four of them into five distinct clusters, suggesting that these four endophytic fungi may possess at least five distinct functional categories of terpene synthases (Fig 2A). The endophytic TPSs were also compared to several plant and fungal TPSs and were found to have low sequence similarity with all the plant TPSs and most of the fungal TPS, except for two uncharacterized putative TPSs from *Trichoderma virens* (EHKY27518) and *Neurospora tetraspema* (EGZ75309) that shared higher sequence similarity with the three endophytic caryophyllene synthases and EC12-GS, respectively, (Fig 2B and 2C). However, due to low sequence similarity to well characterized TPSs (Fig 2B and 2C), it’s difficult to predict function using sequence information alone, necessitating a functional characterization of each putative TPS in order to determine its catalytic activity.

**Table 1:** Protein sequence alignment of the predicted terpene synthases (TPSs) from sequenced four endophytes. Active TPSs are highlighted in bold in the first column.

Protein ID	Aspartate Rich Motif	NSE/DTE Triad	Major Products
**Cluster 1**	DDXXE	NDXXSXXKE	
CI4A-20984	GYWDDLSEKLVSEPTA	IVNDLISFKKEMKA	
CO27-269219	LYWDDLVEGLAHDASA	IVNDLISFKKEMKA	N/A
EC38-3887	LHWDDLVEGLADDPSA	IVNDLISFKKEMKA	
EC12-25458	GYWDDLSESLAVDPVA	IVNDLISFKKEMKA	
**Cluster 2**	DDXXD	NDXXSXXKE	β-cis-ocimene, β-pinene,
CI4A-216497	IFVWDDEVDAGDTDVS	ILNDVYSVQKEIAQ	1S-α-pinene,α-selinene
CO27-31178	IFVWDDEIDAGDTDVS	ILNDVYSVQKEIAQ	α-guaiene, viridiflorol
**EC12-17536**	IFVWDDEVDAGDTDVS	ILNDVYSVQKEIAQ	δ-guaiene, (-)-β-elemene
**EC38-200002**	IFVWDDEIDAGDTDVS	ILNDVYSVQKEIAQ	(-)-alloaromadendrene
**Cluster 3**	DDXXD	NDXXSXXKE	β-caryophyllene, humulen-(V1), (-)-α-neoclovene
**CI4A-6706**	FWDDEIDNGGELTDDE	LLNDVLSLQKEFRVS	α-gurjunene, α-selinene,α-guaiene, (+)-longifolene
**CO27-397991**	FWDDEIDTGGDLTEDE	LLNDVLSLQKEFRVS	thujopsene-i3, β-pinene, α-himachalene, β-cubebene
EC12-24764	FWDDEIDTGGDLTEDR	LLNDVLSFQKEFRVS	τ-gurjunene, δ-elemene, α-caryophyllene
**EC38-373976**	FWDDEIDTGGELTEDE	LLNDVLSLQKEFRVS	1S-α-pinene, aromadendrene, (+)-valencene
**Cluster 4**	DDXXE	NDXXSXXKE	β-chamigrene, β-pinene, α-limonene
**CI4A-322581**	FLIDDLLEYMSLEDGKA	VINDIWSFEKELLASK	2-carene, β-cis-ocimene, (-)-β-elemene
**CO27-392541**	FLIDDLLEHMSLEDGRA	VINDIWSYEKEVLAAQ	4-methyl-3-(1-methylethyldene)-1-cyclohexene
EC12-12539	FLIDDVLEDMSFEEGTA	IMNDIWSFEKEALTAA	(+)-valencene, τ-terpinene, α-gurjunene
**EC38-328361**	FLIDDLLEHMSLEDGRA	VINDIWSYEKEVLAAQ	(-)-alloaromadendrene, β-farnesene
**>EC38-80361**	FLIDDVLENMSFEEGTA	VINDIWSFEKEALTAT	β-caryophyllene, (-)-isoledene, 1S-α-pinene
CI4A-59230	FLIDDLLEEMSFADGEA	IVNDIYSFEKEVIAA	
***Cluster 5***	(D/E)DXXE	XDXXXSXXRE	τ-gurjunene, τ-muurolene
CI4A-323210	VSEHDFENVTKRPRVE	RGCKFLISKQREDGG	β-pinene, τ-elemene, 1S-α-pinene
CO27-37618	ASDDKLETLTKRPRLE	RGCDFLISKQREDGG	α-gurjunene, β-cis-ocimene
EC38-80359	ASDDKLETITKRPRLE	RGCDFLISKQREDGG	α-selinene, (-)-alloaromadendrene
**EC12-315006**	VSEDAPETTVKRPRLE	RGCDFLISKQREDGG	
Non-Clustered	DDXXD	NDXXSXXK(E/D)	α-selinene, (-)-alloaromadendrene,τ-elemene
CI4A-7067	FVWDDETDPDESSAMV	TTNDILSFKKEISQ	β-pinene, β-cubebene, β-cis-ocimene
**EC12-24646**	FYWDDVYDFG---DFND	VTNDIVSARQELQC	α-gurjunene, 1H-cyclopropa-α-naphthalene
CI4A-69724	FLFDDQFDEG---HLKE	LVNDVLSYRKDLEL	(-)-isoledene, β-caryophyllene, L-alloaromadendrene
**EC12-70183**	FLFDDQFDEG---HLKD	LVNDILSYKKDLDL	α-gurjunene, (+)-valencene

### Expression of endophytic TPSs in *E*. *coli*

To determine their function, the 26 predicted TPS genes were codon optimized and expressed in *E*. *coli* along with the geranyl pyrophosphate synthase (GPPS) gene from *Abies grandis* (GenBank: AF513112.1, GPPS_Ag_) and a plasmid harboring the entire mevalonate pathway [35]. This plasmid was used to increase the flux of carbon through the terpene pathway with the aim of enhancing productivity and increasing the chance that even poorly expressed TPS will produce detectable levels of terpenes. The VOC products of each TPS present in the headspace of the culture flask were extracted by SPME and analyzed with GC-MS. Out of the 26 putative endophytic TPSs tested, 12 were active, producing a mixture of mono- (C_10_) and sesquiterpenes (C_15_) (Tables 2 and 3). TPSs in the same cluster tended to produce a similar spectrum of terpene compounds and are therefore discussed by cluster. The profiles of each TPS cluster are summarized in this section and described in detail below. No terpene compounds were produced by the TPSs in the cluster 1, and they are not discussed further. The TPSs in cluster 2 primarily produced monoterpenes, including pinene (1**a**, 1**b**), ocimene (1**c**), and limonene (1**d**), and a lower abundance (<20%) of sesquiterpenes. The TPSs in cluster 3 yielded a wide spectrum of sesquiterpenes and some monoterpenes. Caryophyllene (2**d**, 2**d**, 2**g**) and its isomers were the major product of these enzymes, accounting for up to 80% total peak abundance. The terpene profiles from TPSs in clusters 4 and 5 are less complex than cluster 3 TPSs, and include the sesquiterpenes chamigrene (3**f**), and gurjunene (2**a**, 2**b**). The non-clustered TPS EC12-SS (SS: Selinene Synthase) and EC12-ILS (IsoLedene Synthase) primarily produced the sesquiterpenes selinene (2**h**) and isoledene (5**a**), respectively. The activity of these TPSs correlated well with the terpene products produced by their native hosts. All the major terpenes (pinene, limonene, caryophyllene, chamigrene, gurjunene, selinene, and isoledene) produced from the functional TPSs were detected in the VOC profiles of the four endophytes grown on potato dextrose. The functional endophytic TPSs had low protein sequence similarity compared to other type I TPSs from plants, but retained a conserved DDXXD motif.

**Table 2:** The list and nomenclature of active terpene synthases in this study.

Gene Name	Enzyme Function	JGI Protein ID	Cluster
EC12-PGS	Pinene and Guaiene Synthase	17536	2
EC38-PGS	Pinene and Guaiene Synthase	200002	
CI4A-CS	Caryophyllene Synthase	6706	
CO27-CS	Caryophyllene Synthase	397991	3
EC38-CS	Caryophyllene Synthase	373976	
CI4A-CPS	Chamigrene and Pinene Synthase	322581	
CO27-CPS	Chamigrene and Pinene Synthase	392541	
EC38-CPS	Chamigrene and Pinene Synthase	328361	4
EC38-GPS	Gurjunene and Pinene Synthase	80361	
EC12-GS	Gurnunene Synthase	315006	5
EC12-SS	Selinene Synthase	24646	non
EC12-ILS	IsoLedene Synthase	70183	non

**Table 3:** The most abundant terpene compounds from each cluster and TPS.

**Clusters**	Compounds	EC12-GS, % of total Peak Area			
	**τ-gurnunene**	58.03			
**Cluster 5**	τ-muurolene	3.88			
	*β*-pinene	3.71			
	**α-selinene**	50.71			
**EC12-SS**	(-)-Alloaromadendrene	8.15			
	τ -elemene	6.71			
	**(-)-isoledene**	10.8			
**EC12-ILS**	iso-longifolene	6.76			
	β-caryophyllene	6.71			
		EC12-PGS, % of total Peak Area	EC12-PGS, % of total Peak Area		
	β-pinene	17.64	9.4		
**Cluster 2**	α-pinene	16.92	21.04		
	**β-cis-ocimene**	21.06	44.52		
	α-guaiene	11.03	8.16		
		CI4A-CS, % of total Peak Area	EC38-CS, % of total Peak Area	CO27-CS, % of total Peak Area	
	**caryophyllene-(II)**	12.24	18.1	21.94	
	β-caryophyllene	12.75	13.06	12.46	
**Cluster 3**	α-selinene	6.74	7.59	9.81	
	humulen-(vl)	12.21	5.94	6.71	
		EC38-CPS, % of total Peak Area	CI4A-CPS, % of total Peak Area	EC38-GPS, % of total Peak Area	CO27-CPS, % of total Peak Area
	**β-chamigrene**	24.38	61.28	β-elemene, 4.60	65.35
	α-gurjunene	2-carene, 2.23	β-cis-ocimene, 2.53	20.41	β-elemene, 3.99
**Cluster 4**	α-limonene	10.23	3.8	9.83	3.89
	β-pinene	30.71	16.24	16.36	10.39

An examination of other reports that describe recombinantly expressed TPS indicate that these enzymes tend to produce a single class of terpene, i.e. monoterpenes or sesquiterpenes [1, 13, 37–42]. There are a few reports using *in vitro* assays that show the production of both mono- and sesquiterpenes from high concentrations of GPP or FPP substrates [43]. However, it was never demonstrated that this bi-functionality extends to an *in vivo* activity, so it is unclear whether or not this is a phenomenon that would actually occur in nature. This study is the first to demonstrate that TPSs can be bi-functional *in vivo*, producing both mono- and sesquiterpenes. It could be argued that the *E*. *coli* strain used in this study has artificially altered the levels of GPP and FPP, but several other TPS have been expressed in this strain that do not exhibit this characteristic, and many other recombinant strains also have altered isoprenoid precursor levels and none have had recombinant TPS that exhibit this behavior. Therefore, this phenomenon appears to be enzyme specific. It will be interesting to further investigate these enzymes to identify the structural features that enable this bi-functionality and to determine the impacts of GPP and FPP levels on product distribution. Also, it will be interesting to determine whether or not this is a widespread phenomenon that extends to the other TPS that have exhibited bi-functionality *in vitro*

### Cluster 2: bi-functional α-, β-pinene/*α*-guaiene synthases

**Fig 3:**
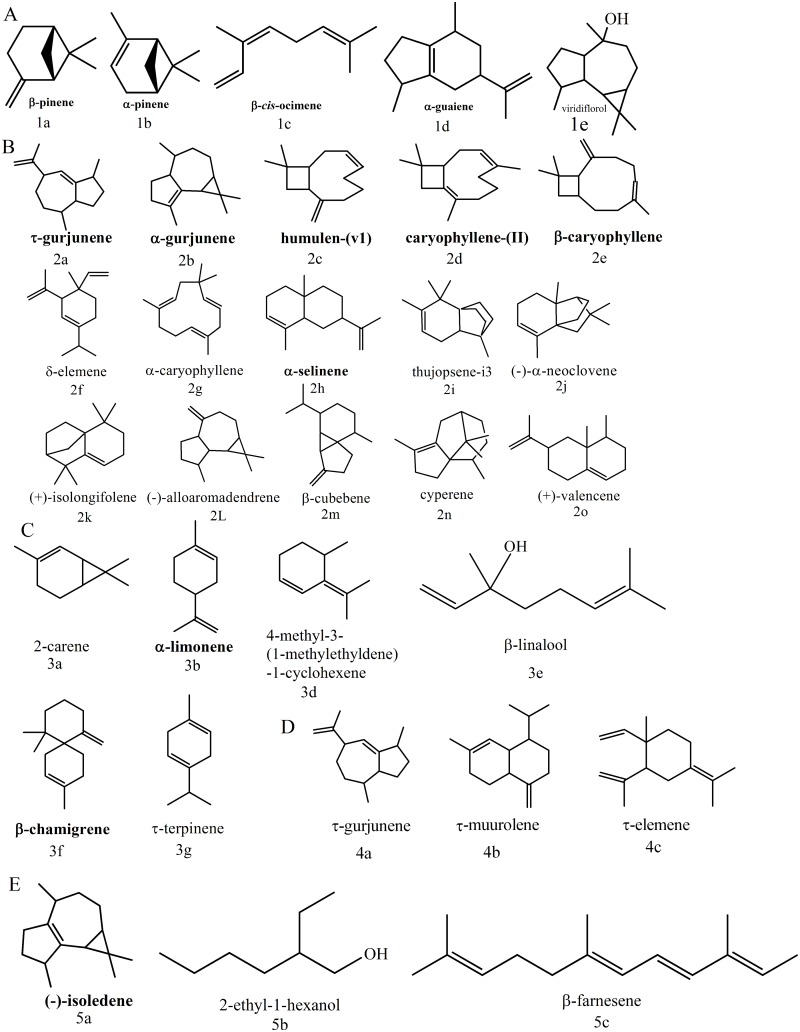
Terpene profiles of TPSs in A) cluster 2, B) cluster 3, C) cluster 4, D) cluster 5, and E) non-clustered TPSs. The number and letter below the structures refers to the compounds in S1–S5 Tables.

In cluster 2, the TPS EC12-PGS(PGS: Pinene and Guaiene Synthase, Tables 2 and 3) from *Daldinia eschscholzii* EC12 and the TPS EC38-PGS from *Hypoxylon sp*. EC38 were active and produced β-*cis*-ocimene (C_10_,**1c**), β-pinene (C_10_, **1a**), and 1s-α-pinene (C_10_, **1b**) as major compounds (Fig 3A, S1 Table). The α-pinene, β-pinene and β-*cis*-ocimene accounted for 55.6% and 75% of total peak area of GC spectra from strains expressing protein EC12-PGS and EC38-PGS, respectively, which indicates that these two enzymes are pinene synthases. Additionally, the existence of two stereoisomeric products of pinene suggests that these two pinene synthases fall into class II pinene cyclases [42, 44]. Supplementary S1 Fig outlines the mechanism of pinene biosynthesis [42]. β-*cis*-ocimene may be the product of deprotonation followed by intramolecular electrophilic attack of a linalyl cation derived from GPP [44] or it is a possible artifact, as it has been reported to form in the injection port of the gas chromatography instrument used to analyze the products via thermal rearrangement of pinene [45]. Further analysis is required to determine whether this is a bona fide TPS product or an artifact. Interestingly, a significant amount of the sesquiterpene α-guaiene (C_15_, **1d**) was also produced by EC12-PGS (11.026% of total peak area) and EC38-PGS (8.16% of total peak area). Other minor products were detected as well, including α-selinene (C_15_, **2h**), alloaromadendrene (C_15_, **2l**) and its oxidation product viridiflorol(**1e**)[46] (Table 1 and S1 Table). The GPPS from *Abies grandis* used in this study was reported to specifically produce GPP, accepting only one DMAPP and one IPP co-substrates [47]. However, the *E*.*coli* strain used in this study harbors a native farnesyl pyrophosphate synthase (FPPS) gene(*ispA*), and is therefore the likely source of the FPP used to synthesize these sesquiterpenes [48]. The production of multiple monoterpenes and sesquiterpenes by these two TPSs indicates that they are bi-functional mono-/sesquiterpene synthases.

To date, the most thoroughly characterized pinene synthases are from plants, including *Pinus taeda*[49], *Abies grandis*[39, 41], *Artemisia annua*[50], *Cannabis sativa*[51], and *Picea abies*[52]. All of these plant pinene synthases have been expressed in *E*.*coli* and none of them can produce sesquiterpenes [41, 49–53]. They primarily produced α-pinene and β-pinene, as well as lower amounts of other monoterpenes such as limonene [39, 41, 49], camphene [39, 41, 49], myrcene [39, 41, 51], and α-terpenolen [51]. A δ-guaiene synthase from the plant *Aquilaria crassna* has also been characterized, and like the plant pinene synthases, it only produced a single class of terpene, including the sesquiterpenes δ-guaiene, α-guaiene, germacrene A, β-elemene, and α-humulene [54–56]. The endophytic pinene/guaiene synthases described in this report have the same DDXXD motif as the plant pinene and guaiene synthases, but otherwise have low sequence similarity to the plant TPSs. There was no obvious structure/function relationship that could be determined that would explain how the endophytic TPSs are bi-functional while the others are not.

### Cluster 3: α-, β-caryophyllene synthases

In cluster 3, only the TPSs CI4A-CS (CS: Caryophyllene Synthase, Tables 2 and 3), CO27-CS, and EC38-CS were active. These three TPSs produced multiple terpenes, including both mono-and sesquiterpenes, with these sesquiterpenes accounting for more than 90% of total peak area. Among these sesquiterpenes, different caryophyllene stereoisomers, such as α-caryophyllene (**2g**), caryophyllene-(II)(**2d**), humulen-(v1) (**2c**), and β-caryophyllene (**2e**)were most abundant, accounting for more than 50% of total sesquiterpene peak area (Fig 3B, S2 Table). Therefore, these three TPSs appear to be primarily caryophyllene synthases. In addition to β-caryophyllene and its stereoisomers, CI4A-CS from *Hypoxylon sp*. CI4A yielded various amounts of other sesquiterpenes, including humulen-(v1) (**2c**), gurjunene (**2a**, **2b**), α-guaiene (**1d**), α-selinene (**2h**), etc., as well as monoterpenes, including β-pinene (**1a**), 1S-α-pinene (**1b**), and β-*cis*-ocimene (**1c**)(Fig 3, S2 Table). EC38-CS and CO27-CS produced a similar array of terpenes, except CO27-CS produced thujopsene-i3(**2i**), α/τ-neoclovene (**2j**) and β-cubebene (**2m**), while EC38-CS produced (-)-α-neoclovene(**2j**), β-cubebene(**2m**), and (+)-longifolene(**2k**). The production of multiple terpenes by each of these enzymes is likely due to various cyclization reactions of different intermediate carbocations that are formed by intramolecular electrophilic attacks and deprotonations of FPP [44, 57]. Supplementary S3 Fig outlines a possible reaction mechanism of the formation of some of these terpenes.

There are no reports of fungal caryophyllene synthase, but there are several caryophyllene synthases from plants: cotton [58], *Artimisia annua*[59], maize [60], rice [61], and *arabidopsis*[62]. The plant caryophyllene synthases from cotton, maize, and *arabidopsis* use FPP as a substrate, rather than GPP, and produced several sesquiterpene compounds, including β-caryophyllene [58, 60, 62], α-humulene [58, 62], (-)-α-copaene [62], linalool [60], 4,8-dimethylnona-1,3,7-triene [60], (E)-α-bergamotene [60], and (E)-β-farnesene [60]. The caryophyllene synthase from rice has similar substrate specificity, but produced more than 25 different sesquiterpene compounds, consisting of β-caryophyllene, β-farnesene, α-bergamotene, β-elemene, etc. All the caryophyllene synthases from plants share the same DDXXD motif with the endophytic TPSs, but have different NSE/DTE triad amino acid sequences and low protein sequence similarity. As with cluster 2 TPSs, the cluster 3 caryophyllene synthases are bi-functional, producing both mono-and sesquiterpenes.

### Cluster 4: bi-functional β-chamigrene/β-pinene and α-gurjunene/β-pinene synthases

In cluster 4, the TPSs CI4A-CPS (CPS: Chamigrene and Pinene Synthase, Tables 2 and 3), CO27-CPS, EC38-CPS, and EC38-GPS (GPS: Gurjunene and Pinene Synthase, Tables 2 and 3) were functional. The TPSs CI4A-CPS, CO27-CPS, and EC38-CPS produced similar terpenes, while EC38-GPS had a distinct profile. All four TPSs produced both monoterpenes and sesquiterpenes, indicating that, again, they are bi-functional mono-/sesquiterpene synthases. The TPSs CI4A-CPS, CO27-CPS, and EC38-CPS, produced β-chamigrene (C_15_, **3f**) as the major product (>34.8%), indicating that these three enzymes are primarily chamigrene synthases (Fig 3C, S2 Table). S3 Fig outlines a possible reaction mechanism for the biosynthesis of β-chamigrene. These three enzymes also produced lower amounts monoterpenes: β-pinene (**1a**), α-limonene (**1d**), β-*cis*-ocimene (**1c**), 4-methyl-3-(1-methylethylidene)-1-cyclohexene(**3d**), and α-pinene(**1b**), with β-pinene being the major monoterpene in each case (Table 1, S3 Table). Other minor products include2-carene (EC38-CPS, CI4A-CPS, **3a**), (-)-β-elemene (EC38-CPS, CO27-CPS, **2f**),(+)-valencene (CO27-CPS, **2o**), and τ-terpinene (CO27-CPS, **3g**). The mechanism for the biosynthesis of α-limonene, 2-carene, and τ-terpinene is represented in S1 Fig. There are no reports of a TPS producing chamigrene as the sole sesquiterpene. However, Shuiqin Wu et al. reported that an α-barbatene synthase from *Arabidopsis* produced a mix of α-barbatene, thujopsene, and β-chamigrene as major products, but no monoterpenes [63]. The endophyte TPSs share low sequence similarity with the plant chamigrene synthase, have the same DDXXD motif, and a different NSE/DTE triad.

The fourth TPS in cluster 4, EC38-GPS, produced both mono- and sesquiterpenes, a common theme with the endophyte TPSs in this report. The monoterpene β-pinene (**1a**) and sesquiterpene α-gurjunene (**2b**) are the two major products of this enzyme, accounting for 16.4% and 20.4% of total peak area, respectively. Minor products include, α-limonene (**3b**), β-elemene (**2f**), L-alloaromadendrene (**2l**), β-*cis*-ocimene (**1c**), α-pinene (**1a**), β-farnesene (**5c**), β-caryophyllene (**2d**), (-)-isoledene (**5a**), and 4-methyl-3-(1-methylethylidene)-1-cyclohexene (**3d**). Schmidt et al.[64] discovered an α-gurjunene synthase from *Solidago canadensis*, which produced germacrene D (50%), α-gurjunene (42%), γ-gurjunene (4%) as major products, but no monoterpenes. It has low sequence identity with EC38-GPS, so it is difficult to predict why these enzymes have different functionality based solely on sequence. This is true even within the cluster 4; EC38-GPS has low sequence similarity relative to the other three TPSs in cluster 4, but still clusters with them, indicating that the differences in sequence that dictate different function are subtle and will require extensive analysis to uncover.

### Cluster 5: τ-gurjunene synthase

EC12-GS (GS: Gurjunene Synthase, Tables 2 and 3) was the only active TPS in cluster 5. Its major product was the sesquiterpene τ-gurjunene (**4a**, 58.7% of total peak area), suggesting that it is primarily a τ-gurjunene synthase (Fig 3; Table 1 and S4 Table). EC12-GS has a broader product profile compared to the α-gurjunene synthase from *Solidago Canadensis*[64], which only produced three sesquiterpenes as major products. Although gurjunene accounted for ~60% of the terpenes produced by EC12-GS, it also produced the sesquiterpenes τ-muurolene (**4b**), τ-elemene (**4c**), and the monoterpenes β-pinene (**1a**), α-pinene (**1b**), and β-*cis*-ocimene (**1c**), indicating this enzyme is a bi-functional mono-/sesquiterpene synthase. The possible mechanism of the τ-gurjunene formation is outlined in S2 Fig.

### Unclustered TPS: α-selinene and (-)-isoledene synthases

The putative TPSs EC12-SS (SS: Selinene Synthase) and EC12-ILS (ILS: IsoLedene Synthase) shared low sequence homology with the other predicted endophytic TPSs and did not cluster. EC12-SS produced multiple mono- and sesquiterpenes, but α-selinene (**2h**) was the major terpene produced (50.7% of total peak area), which suggests that this enzyme is primarily a selinene synthase (Fig 3; Tables 2 and 3, S5 Table). Steele et al. reported the discovery of a δ-selinene synthase (*ag*4) from *Abies grandis*[13] that produced more than 20 sesquiterpenes including δ-selinene (25.3%), (E,E)-germacrene B (17.4%), guaia-6,9-diene(9.7%), germacrene A (6.7%), δ-amorphene (6.4%), germacrene C (3.4%), α-selinene (1.7%), β-caryophyllene (1.5%), δ-cadinene (1.4%), seli-3,7(11)-diene (1.2%), etc. Compared to this plant δ-selinene synthase, EC12-SS yielded fewer sesquiterpenes and a higher relative abundance of α-selinene (50.8%). It is also a bi-functional mono-/sesquiterpene synthase (S5 Table). The proposed mechanism for selinene biosynthesis is in S3 Fig.

In contrast to most of the other endophyte TPS characterized, EC12-ILS produced only sesquiterpenes, with (-)-isoledene (**5a**) being the most abundant at 10.8% of the total peak area. The other terpenes produced include iso-longifolene (2**k**) and β-caryophyllene (2**d**), accounting for 12.5% of total peak area. There have been other studies that reported the detection of isoledene [10–12] in plants, and a putative isoledene synthase was predicted in the genome of *Eucalyptus grandis*[65]. However, EC12-ILS is the first isoledene synthase enzyme to be functionally characterized.

### Potential applications for endophyte-derived monoterpenes and sesquiterpenes

Next generation biofuels are expected to have high energy density and physicochemical properties compatible with current engine design, transportation systems, and storage infrastructure. Hydrocarbons derived from terpenes meet most of these criteria as they are structurally similar to the compounds in petroleum distillate fuels, and often have similar combustion properties [66]. For example, hydrogenated pinene (C_10_) dimers were reported to contain high volumetric energy similar to that of jet fuel JP-10 [67]. The hydrogenated product of the sesquiterpene bisabolene(C_15_) was shown to have better properties than D2 diesel, such as lower cloud point, and higher flash point and API gravity [14]. The most abundant terpenes produced in this study are pinenes and sesquiterpenes, such as guaiene, caryophyllene, chamigrene, gurjunene, and selinene. These terpenes are hydrocarbons or hydrocarbon-like compounds with a carbon content in the C_10_ and C_15_ range and are therefore good candidates for “drop-in” aviation fuels. Simultaneous satisfaction of combustion specifications and specifications for physical properties such as density, energy content, and viscosity often require blending of different types of hydrocarbons. The use of terpenes and terpene derivatives as blendstocks for renewable fuels for aviation and diesel applications has recently been discussed by Harvey et al.[68], who determined that blending hydrogenated sesquiterpenes with synthetic branched paraffins could raise cetane numbers and reduce viscosity, producing biosynthetic fuels that meet applicable jet and diesel specifications.

In addition to their potential use as biofuels, most of the terpenes reported here are major components of essential oils used in the fragrance and flavoring industries (α-guaiene, β-chamigrene, α-gurjunene, etc.) and many have potential pharmaceutical applications. For example, it has been reported that pinene can act as an anti-tumor [69, 70], and anti-repression [71] agent. Also, the sesquiterpene caryophyllene not only has been considered one of the top three most promising high energy “drop in” jet fuels [72] it also has multiple potential pharmaceutical applications, such as anti-cancer [73], anti-inflammatory [73–75], life-span elongation [76], neuroprotection [77], insulin secretion moderation [78], acute and chronic pain attenuation [79], and alcohol dependency release [80].

## Conclusion

Previously, GC-MS was used to analyze the VOCs produced by four fungal endophytes (*Hypoxylon sp*. EC38, CI4A, CO27, and *Daldinia eschscholzii* EC12) and hundreds of terpene compounds were detected [2, 9, 81, 82]. However, most of the TPS enzymes that synthesize these compounds have not been identified. In this study, we leveraged an *E*. *coli* strain harboring a synthetic mevalonate pathway for enhanced terpene production as a synthetic biology platform to screen 26 putative TPSs from these four fungi. The TPSs were identified and characterized by a combination of genomic data mining, phylogenetic analysis, protein sequence alignment, fast products extraction with SPME, and rapid chemical characterization with GC-MS. This approach avoids time-consuming and challenging conventional enzyme discovery routes, such as functional genomics library construction and screening, or biochemical purification of native enzymes, in addition to specific challenges for TPS enzymes, such as terpene compound purification and identification, and thereby establishes a valuable and rapid process for novel TPS discovery. Using this approach, we discovered 12 novel TPSs clustered into 4 homology groups that have potential uses in medicine and other industries, including the nascent biofuels sector [83].

## Supporting Information

S1 Fig Mechanism for the biosynthesis of monoterpenes: α-, and β-pinene, α-limonene, 2-careen, β-ocimene, and τ-terpinene. The biosynthesis of pinene can be rationalized by postulating that GPP ionizes to a stable allylic cation, followed by collapse to linalyl diphosphate (LPP). The reionization of the LPP cisoid conformer followed by intramolecular electrophilic addition generates the transient α-terpinyl cation. Alternatively, an additional electrophilic attack on the newly formed cyclohexenoid double bond of α-terpinyl cation generates the pinane skeleton, which deprotonated by terpene cyclase II to form both α- and β-pinene.
(DOCX)

S2 Fig Mechanism for the biosynthesis of sesquiterpenes: α-, and β-caryophyllene, humulen, α-selinene, α-guaiene, and τ-gurjunene. Generally, FPP is ionized to generate an allylic cation, and then through a 11,1 closure to form humulyl cation and a subsequent deprotonation to yield α-caryophyllene, or through a 11,1 closure to form humulyl cation, another 2,10 closure to generate caryophylylcation and further deprotonated to form humulen-(v1) and β-caryophyllene. Also, FPP can be ionized and through a subsequent 10,1 closure and deprotonation, form germacrene A, which can be an intermediate for further intramolecular electrophilic attack, hydride shift, and deprotonation, yield α-selinene, α-guaiene, and τ-gurjunene.
(DOCX)

S3 Fig Mechanism for the biosynthesis of sesquiterpenes β-chamigrene and thujopsene. Chamigrene biosynthesis could begin with the ionization and subsequent allylic rearrangement of the diphosphate moiety of FPP, allowing for the formation of nerodidyl diphosphate (NPP, cisoid conformation). Reionization of the ciscoid conformation of NPP and subsequent intramolecular electrophilic attack would form a bisabolyl cation, which followed by a secondary intramolecular electrophilic attack and 1,4-hydride shift, would create a cuprenyl cation. A subsequent methylene migration would yield the chamigrenyl cation which could undergo a direct proton abstraction to form β-chamigrene.
(DOCX)

S1 Table Terpene profiles of TPS in the cluster 2. (DOCX)

S2 Table Terpene profiles of TPS in the cluster 3. (DOCX)

S3 Table Terpene profiles of TPS in the cluster 4. (DOCX)

S4 Table Terpene profiles of TPS in the cluster 5. (DOCX)

S5 Table Terpene profiles of TPS in non-clustered group. (DOCX)

S6 Table Compound profile of the control strain lacking TPS. (DOCX)

S7 Table GC spectrum of terpene standards. (DOCX)
