# Supplementary material for: Rapid Discovery and Functional Characterization of Terpene Synthases from Four Endophytic Xylariaceae
Source: PLoS One. 2016 Feb 17;11(2):e0146983. doi: 10.1371/journal.pone.0146983 (PMC4757406; doi:10.1371/journal.pone.0146983)
Supplement: S1 Fig — The biosynthesis of pinene can be rationalized by postulating that GPP ionizes to a stable allylic cation, followed by collapse to linalyl diphosphate (LPP). The reionization of the LPP cisoid conformer followed by intramolecular electrophilic addition generates the transient α-terpinyl cation. Alternatively, an additional electrophilic attack on the newly formed cyclohexenoid double bond of α-terpinyl cation generates the pinane skeleton, which deprotonated by terpene cyclase II to form both α- and β-pinene. (DOCX) [file pone.0146983.s001.docx]

Rapid Discovery and Functional Characterization of Terpene Synthases from Four Endophytic Xylariaceae

Weihua Wu^1^, William Tran^1^, Craig A. Taatjes^2^, Jorge Alonso-Gutierrez^3,4^, Taek Soon Lee^3,4^, John M. Gladden^1,4,^*
^1^ Biomass Science & Conversion Technologies, Sandia National Laboratories, Livermore, CA, USA ^2^Combustion Chemistry Department, Sandia National Laboratories, Livermore, CA, USA; ^3^Physical Biosciences Division, Lawrence Berkeley National Laboratory, Berkeley, CA, USA; ^4^Joint BioEnergy Institute, Emeryville, CA, USA

Supplemental Data

**Figure S1.** Mechanism for the biosynthesis of monoterpenes: α-, and β-pinene, α-limonene, 2-careen, β-ocimene, and τ-terpinene[[1](#_ENREF_1), [2](#_ENREF_2)]. The biosynthesis of pinene can be rationalized by postulating that GPP ionizes to a stable allylic cation, followed by collapse to linalyl diphosphate (LPP). The reionization of the LPP cisoid conformer followed by intramolecular electrophilic addition generates the transient α-terpinyl cation. Alternatively, an additional electrophilic attack on the newly formed cyclohexenoid double bond of α-terpinyl cation generates the pinane skeleton, which deprotonated by terpene cyclase II to form both α- and β-pinene[[3](#_ENREF_3)]

1. Edward M. Davis RC: **Cyclization enzymes in the biosynthesis of monoterpenes, sesquiterpenes, and diterpenes**. *Topics in Current Chemistry* 2000, **209**:53-95.

2. Dewick PM: **The biosynthesis of C_5_-C_25_ terpenoid compounds**. *Natural Product Reports* 2002,1**9**:181-222.

3. Croteau HGaR: **Pinene Cyclases I and II. Two enzymes from SAGE (*Salvia Officinalis*) which catalyze stereospecific cyclizations of geranyl pyrophosphate to monoterpene olefins of opposite configuration**. *The Journal of Biological Chemistry* 1984, **259**(2):740-748.
