# Supplementary material for: Rapid Discovery and Functional Characterization of Terpene Synthases from Four Endophytic Xylariaceae
Source: PLoS One. 2016 Feb 17;11(2):e0146983. doi: 10.1371/journal.pone.0146983 (PMC4757406; doi:10.1371/journal.pone.0146983)
Supplement: S2 Fig — Generally, FPP is ionized to generate an allylic cation, and then through a 11,1 closure to form humulyl cation and a subsequent deprotonation to yield α-caryophyllene, or through a 11,1 closure to form humulyl cation, another 2,10 closure to generate caryophylylcation and further deprotonated to form humulen-(v1) and β-caryophyllene. Also, FPP can be ionized and through a subsequent 10,1 closure and deprotonation, form germacrene A, which can be an intermediate for further intramolecular electrophilic attack, hydride shift, and deprotonation, yield α-selinene, α-guaiene, and τ-gurjunene. (DOCX) [file pone.0146983.s002.docx]

Rapid Discovery and Functional Characterization of Terpene Synthases from Four Endophytic Xylariaceae

Weihua Wu^1^, William Tran^1^, Craig A. Taatjes^2^, Jorge Alonso-Gutierrez^3,4^, Taek Soon Lee^3,4^, John M. Gladden^1,4,^*
^1^ Biomass Science & Conversion Technologies, Sandia National Laboratories, Livermore, CA, USA ^2^Combustion Chemistry Department, Sandia National Laboratories, Livermore, CA, USA; ^3^Physical Biosciences Division, Lawrence Berkeley National Laboratory, Berkeley, CA, USA; ^4^Joint BioEnergy Institute, Emeryville, CA, USA

Supplemental Data

**Figure S2.** Mechanism for the biosynthesis of sesquiterpenes: α-, and β-caryophyllene, humulen, α-selinene, α-guaiene, and τ-gurjunene[[1](#_ENREF_1), [2](#_ENREF_2)]. Generally, FPP is ionized to generate an allylic cation, and then through a 11,1 closure to form humulyl cation and a subsequent deprotonation to yield α-caryophyllene, or through a 11,1 closure to form humulyl cation, another 2,10 closure to generate caryophylylcation and further deprotonated to form humulen-(v1) and β-caryophyllene. Also, FPP can be ionized and through a subsequent 10,1 closure and deprotonation, form germacrene A, which can be an intermediate for further intramolecular electrophilic attack, hydride shift, and deprotonation, yield α-selinene, α-guaiene, and τ-gurjunene.

1. Dewick PM: **The biosynthesis of C_5_-C_25_ terpenoid compounds**. *Natural Product Reports* 2002, **19**:181-222.

2. Edward M. Davis RC: **Cyclization enzymes in the biosynthesis of monoterpenes, sesquiterpenes, and diterpenes**. *Topics in Current Chemistry* 2000, **209**:53-95.
