# Supplementary material for: Rapid Discovery and Functional Characterization of Terpene Synthases from Four Endophytic Xylariaceae
Source: PLoS One. 2016 Feb 17;11(2):e0146983. doi: 10.1371/journal.pone.0146983 (PMC4757406; doi:10.1371/journal.pone.0146983)
Supplement: S3 Fig — Chamigrene biosynthesis could begin with the ionization and subsequent allylic rearrangement of the diphosphate moiety of FPP, allowing for the formation of nerodidyl diphosphate (NPP, cisoid conformation). Reionization of the ciscoid conformation of NPP and subsequent intramolecular electrophilic attack would form a bisabolyl cation, which followed by a secondary intramolecular electrophilic attack and 1,4-hydride shift, would create a cuprenyl cation. A subsequent methylene migration would yield the chamigrenyl cation which could undergo a direct proton abstraction to form β-chamigrene. (DOCX) [file pone.0146983.s003.docx]

Rapid Discovery and Functional Characterization of Terpene Synthases from Four Endophytic Xylariaceae

Weihua Wu^1^, William Tran^1^, Craig A. Taatjes^2^, Jorge Alonso-Gutierrez^3,4^, Taek Soon Lee^3,4^, John M. Gladden^1,4,^*
^1^ Biomass Science & Conversion Technologies, Sandia National Laboratories, Livermore, CA, USA ^2^Combustion Chemistry Department, Sandia National Laboratories, Livermore, CA, USA; ^3^Physical Biosciences Division, Lawrence Berkeley National Laboratory, Berkeley, CA, USA; ^4^Joint BioEnergy Institute, Emeryville, CA, USA

Supplemental Data

**Figure S3.** Mechanism for the biosynthesis of sesquiterpenes β-chamigrene and thujopsene[[1](#_ENREF_1), [2](#_ENREF_2)]. Chamigrene biosynthesis could begin with the ionization and subsequent allylic rearrangement of the diphosphate moiety of FPP, allowing for the formation of nerodidyl diphosphate (NPP, cisoid conformation). Reionization of the ciscoid conformation of NPP and subsequent intramolecular electrophilic attack would form a bisabolyl cation, which followed by a secondary intramolecular electrophilic attack and 1,4-hydride shift, would create a cuprenyl cation. A subsequent methylene migration would yield the chamigrenyl cation which could undergo a direct proton abstraction to form β-chamigrene[[3](#_ENREF_3)]

1. Edward M. Davis RC: **Cyclization enzymes in the biosynthesis of monoterpenes, sesquiterpenes, and diterpenes**. *Topics in Current Chemistry* 2000, **209**:53-95.

2. Shuiqin Wu MAS, Bryan T. Greenhagen,Shunji Takahashi, Sungbeom Lee, Robert M. Coates, and Joseph Chappell: **Surrogate Splicing for Functional Analysis of Sesquiterpene Synthase Genes**. *Plant Physiology* 2005, **138**:1322-1333.

3. Lin PP, Rabe KS, Takasumi JL, Kadisch M, Arnold FH, Liao JC: **Isobutanol production at elevated temperatures in thermophilic Geobacillus thermoglucosidasius**. *Metabolic Engineering* 2014, **24**:1-8.
