# Supplementary material for: Rapid Discovery and Functional Characterization of Terpene Synthases from Four Endophytic Xylariaceae
Source: PLoS One. 2016 Feb 17;11(2):e0146983. doi: 10.1371/journal.pone.0146983 (PMC4757406; doi:10.1371/journal.pone.0146983)
Supplement: S1 Table — (DOCX) [file pone.0146983.s004.docx]

Rapid Discovery and Functional Characterization of Terpene Synthases from Four Endophytic Xylariaceae

Weihua Wu^1^, William Tran^1^, Craig A. Taatjes^2^, Jorge Alonso-Gutierrez^3,4^, Taek Soon Lee^3,4^, John M. Gladden^1,4,^*
^1^ Biomass Science & Conversion Technologies, Sandia National Laboratories, Livermore, CA, USA ^2^Combustion Chemistry Department, Sandia National Laboratories, Livermore, CA, USA; ^3^Physical Biosciences Division, Lawrence Berkeley National Laboratory, Berkeley, CA, USA; ^4^Joint BioEnergy Institute, Emeryville, CA, USA

Supplemental Data

**Table S1.**

| **TPS EC12-PGS from *Daldinia eschscholzii* EC12** | | | | |
| --- | --- | --- | --- | --- |
| Compound | Retention Time (min) | % total peak area | Match (%)^a^ | R-match (%)^b^ |
| ***β*-*cis*-Ocimene** (**1c**) | 9.524 | **21.06** | 94.7 | 96 |
| ***β*-pinene** (**1a**) | 7.994 | **17.64** | 92.5 | 93.3 |
| **1S-*α*-pinene** (**1b**) | 9.225 | **16.92** | 94.3 | 96.7 |
| **α-guaiene** (**1d**) | 16.482 | **11.03** | 92.1 | 93.7 |
| Viridiflorol (**1e**) | 21.269 | 2.385 | 88.1 | 92.6 |
| **TPS EC38-PGS from *Hypoxylon sp*. EC38** | | | | |
| Compound | Retention Time (min) | % total peak area | Match (%) | R-match (%) |
| ***β*-*cis*-Ocimene (1c)** | 9.56 | **44.52** | 93.3 | 94.8 |
| **1S-*α*-pinene (1b)** | 9.259 | **21.04** | 95 | 96.7 |
| ***β*-pinene (1a)** | 8.041 | **9.40** | 93.9 | 94.5 |
| **α-guaiene (1d)** | 16.49 | **8.156** | 92.4 | 93.7 |
| Viridiflorol (**1e**) | 21.269 | 2.076 | 89.3 | 92.6 |

a) Match: the match factor was obtained by matching all peaks in the sample spectrum with peaks in the library. The match factor provides a sense of spectral similarity between peaks from the sample and peaks from the library, b) R-match: the reverse match value was obtained by ignoring all peaks that were in the sample spectrum but not in the library spectrum. The percentage value presented represents the degree of similarity between the peaks from sample and peaks from the library.


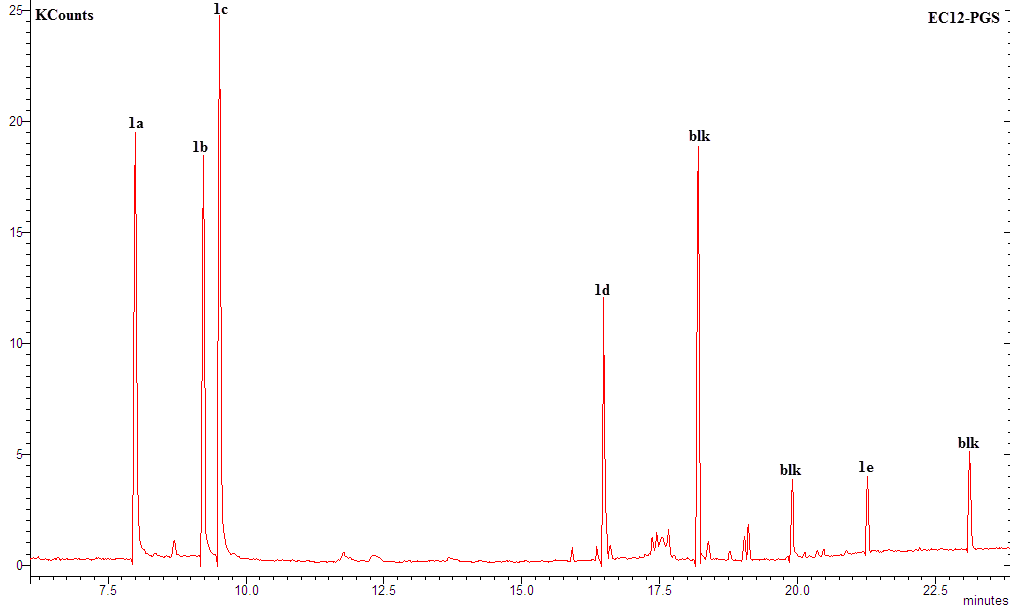


**A**


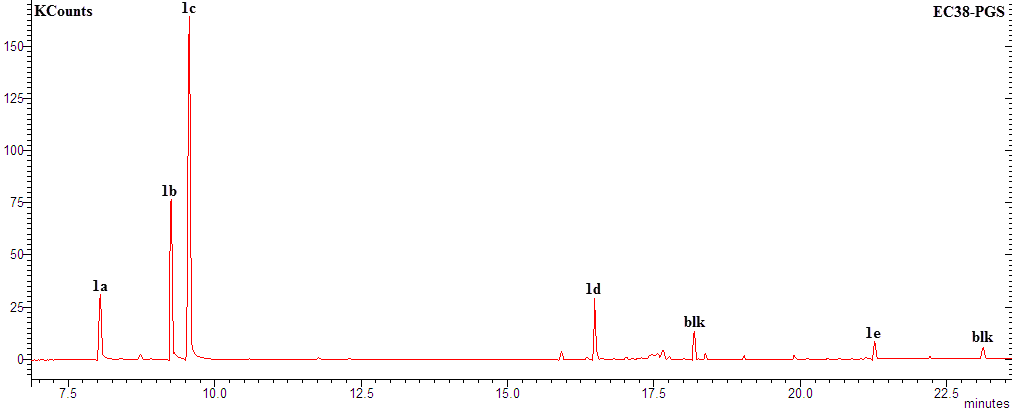


**B**
