# Supplementary material for: Rapid Discovery and Functional Characterization of Terpene Synthases from Four Endophytic Xylariaceae
Source: PLoS One. 2016 Feb 17;11(2):e0146983. doi: 10.1371/journal.pone.0146983 (PMC4757406; doi:10.1371/journal.pone.0146983)
Supplement: S2 Table — (DOCX) [file pone.0146983.s005.docx]

Rapid Discovery and Functional Characterization of Terpene Synthases from Four Endophytic Xylariaceae

Weihua Wu^1^, William Tran^1^, Craig A. Taatjes^2^, Jorge Alonso-Gutierrez^3,4^, Taek Soon Lee^3,4^, John M. Gladden^1,4,^*
^1^ Biomass Science & Conversion Technologies, Sandia National Laboratories, Livermore, CA, USA ^2^Combustion Chemistry Department, Sandia National Laboratories, Livermore, CA, USA; ^3^Physical Biosciences Division, Lawrence Berkeley National Laboratory, Berkeley, CA, USA; ^4^Joint BioEnergy Institute, Emeryville, CA, USA

Supplemental Data

**Table S2.**

| **TPS CI4A-CS from *Hypoxylon sp*. CI4A** | | | | | | |
| --- | --- | --- | --- | --- | --- | --- |
| Compound | Retention Time (min) | | % total peak area | | Match (%) | R-match (%) |
| **caryophyllene-(II) (2d)** | 14.588 | | **13.24** | | 88.1 | 88.5 |
| **Humulene-(V1) (2c)** | 14.344 | | **12.21** | | 90.5 | 90.7 |
| **α-selinene (2h)** | 17.897 | | **6.74** | | 91.7 | 93.2 |
| **β-caryophyllene (2e)** | 15.026 | | **6.38** | | 92.9 | 93.4 |
| **α-guaiene (1d)** | 13.18 | | **5.16** | | 91.7 | 91.8 |
| **α-gurjunene (2b)** | 12.606 | | **5.08** | | 88 | 88.3 |
| Thujopsene-i3 (**2i**) | 21.302 | | 4.07 | | 88.1 | 89.7 |
| β-caryophyllene (**2e1**) | 16.610 | | 4.03 | | 94 | 94.4 |
| α-gurjunene (**2b1**) | 14.062 | | 3.71 | | 89.1 | 90.8 |
| α-gurjunene (**2b2**) | 15.423 | | 3.56 | | 91.2 | 93.5 |
| *β*-pinene (**1a**) | 7.995 | | 3.48 | | 92.3 | 92.7 |
| β-caryophyllene (**2e2**) | 17.282 | | 2.34 | | 94.3 | 94.5 |
| τ-gurjunene (**2a1**) | 13.478 | | 1.84 | | 89.5 | 90.6 |
| δ-elemene (**2f**) | 15.83 | | 1.78 | | 85.5 | 90.1 |
| α-caryophyllene (**2g**) | 17.219 | | 1.67 | | 94.3 | 94.5 |
| τ-gurjunene (**2a**) | 12.263 | | 1.47 | | 90.5 | 92 |
| 1S-*α*-pinene (**1b**) | 9.224 | | 1.12 | | 94.7 | 95.9 |
| *β*-*cis*-Ocimene (**1c**) | 9.552 | | 0.56 | | 91.0 | 91.4 |
| **TPS EC38-CS from *Hypoxylon sp*. EC38** | | | | | | |
| Compound | | Retention Time (min) | | % total peak area | Match (%) | R-match (%) |
| **caryophyllene-(II) (2d)** | | 14.604 | | **18.10** | 89.2 | 89.5 |
| **Thujopsene-i3 (2i)** | | 21.308 | | **10.18** | 89.4 | 91.5 |
| **α-selinene (2h)** | | 17.898 | | **7.59** | 92.6 | 93.5 |
| **Humulene-(V1) (2c)** | | 14.352 | | **5.94** | 90.8 | 91.2 |
| (-)-α-neoclovene (**2j**) | | 14.074 | | 4.76 | 90 | 90.2 |
| β-caryophyllene (**2e**) | | 15.034 | | 4.69 | 93.2 | 93.8 |
| *β*-pinene (**1a**) | | 7.997 | | 4.36 | 92.9 | 93.5 |
| α-gurjunene (**2b2**) | | 15.426 | | 3.50 | 93.1 | 94.2 |
| β-caryophyllene (**2e1**) | | 16.609 | | 3.21 | 94.5 | 94.8 |
| β-caryophyllene (**2e2**) | | 16.735 | | 2.71 | 93 | 93.3 |
| α-gurjunene (**2b**) | | 12.612 | | 2.5 | 88.6 | 89 |
| β-caryophyllene (**2e3**) | | 17.284 | | 2.45 | 93.8 | 94 |
| τ-gurjunene (**2a1**) | | 13.485 | | 2.39 | 89.3 | 90.5 |
| (+)-longifolene (**2k**) | | 15.165 | | 2.37 | 83 | 83.3 |
| τ-gurjunene (**2a**) | | 12.275 | | 1.83 | 90.1 | 91.7 |
| α-caryophyllene (**2g1**) | | 17.22 | | 1.73 | 93.5 | 94.5 |
| α-guaiene (**1d**) | | 13.184 | | 1.41 | 90.3 | 90.8 |
| 1S-*α*-pinene (**1b**) | | 9.229 | | 1.34 | 92.6 | 96.4 |
| (-)-alloaromadendrene (**2l**) | | 15.952 | | 1.24 | 88.7 | 90.2 |
| β-cubebene (**2m**) | | 17.54 | | 1 | 92.7 | 95.3 |
| *β*-*cis*-Ocimene (**1c**) | | 9.527 | | 0.40 | 88.4 | 91 |
| **TPS CO27-CS from *Hypoxylon sp*. CO27** | | | | | | |
| Compound | | Retention Time (min) | | % total peak area | Match (%) | R-match (%) |
| **caryophyllene-(II) (2d)** | | 14.598 | | **21.94** | 88.4 | 89 |
| **α-selinene (2h)** | | 17.899 | | **9.81** | 90.7 | 91.8 |
| **Humulene-(V1) (2c)** | | 14.346 | | **6.71** | 91.5 | 92.1 |
| **β-caryophyllene (2e)** | | 15.027 | | **5.61** | 92.9 | 93.4 |
| Thujopsene-i3 (**2i**) | | 21.303 | | 4.43 | 89 | 90.4 |
| *β*-pinene (**1a**) | | 7.993 | | 4.32 | 93.1 | 93.5 |
| Cyperene (**2n**) | | 14.068 | | 4.15 | 90 | 91 |
| α-gurjunene (**2b2**) | | 15.423 | | 3.92 | 93.5 | 95.1 |
| β-caryophyllene (**2e1**) | | 16.609 | | 3.70 | 93.8 | 94.2 |
| alloaromadendrene (**2l1**) | | 16.736 | | 3.17 | 92.7 | 93 |
| β-caryophyllene (**2e2**) | | 17.283 | | 3.15 | 94.3 | 94.5 |
| α-gurjunene (**2b**) | | 12.605 | | 3.07 | 88.7 | 89 |
| (+)-longifolene (**2k**) | | 15.159 | | 2.61 | 81.7 | 82.9 |
| α-guaiene (**1d1**) | | 13.477 | | 2.48 | 89.3 | 89.9 |
| α-caryophyllene (**2g1**) | | 17.22 | | 2.12 | 93.8 | 95 |
| α-guaiene (**1d**) | | 13.177 | | 1.49 | 89.8 | 90.7 |
| β-cubebene (**2m**) | | 17.539 | | 1.27 | 90.9 | 94 |
| (-)-alloaromadendrene (**2l**) | | 15.95 | | 1.26 | 89.4 | 90.7 |
| (+)-Valencene (**2o**) | | 16.428 | | 1.24 | 91.1 | 92.8 |
| 1S-*α*-pinene (**1b**) | | 9.222 | | 1.21 | 93.4 | 96.8 |
| τ-gurjunene (**2a**) | | 12.268 | | 0.85 | 89.5 | 91 |
| *β*-*cis*-Ocimene (**1c**) | | 9.522 | | 0.51 | 88.2 | 91.5 |


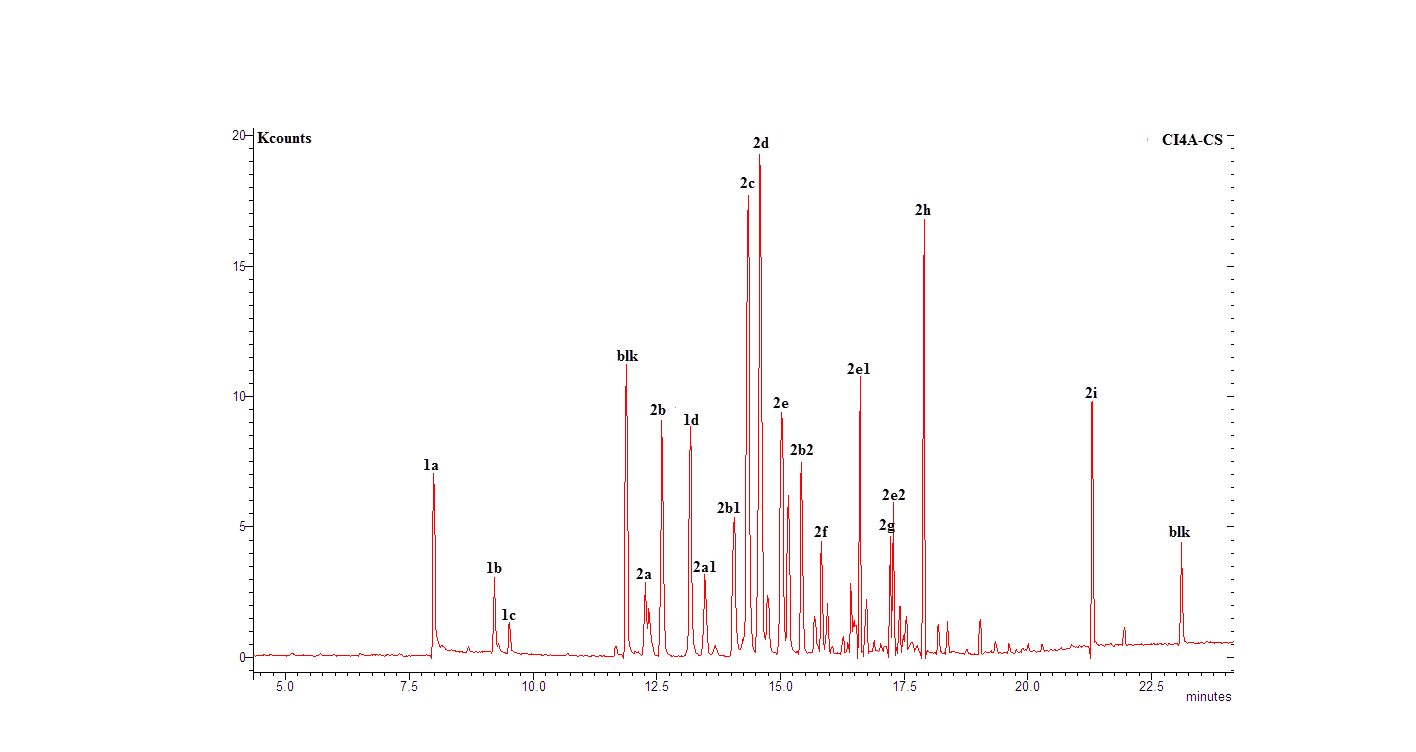


**A**


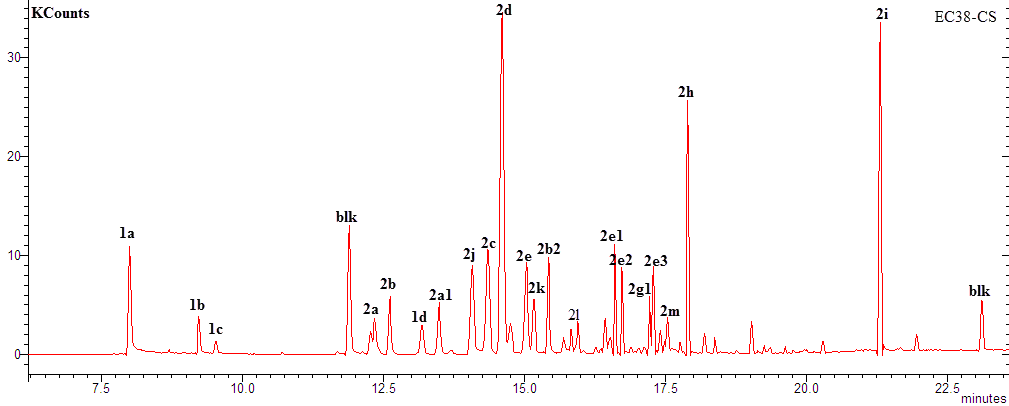


**B**


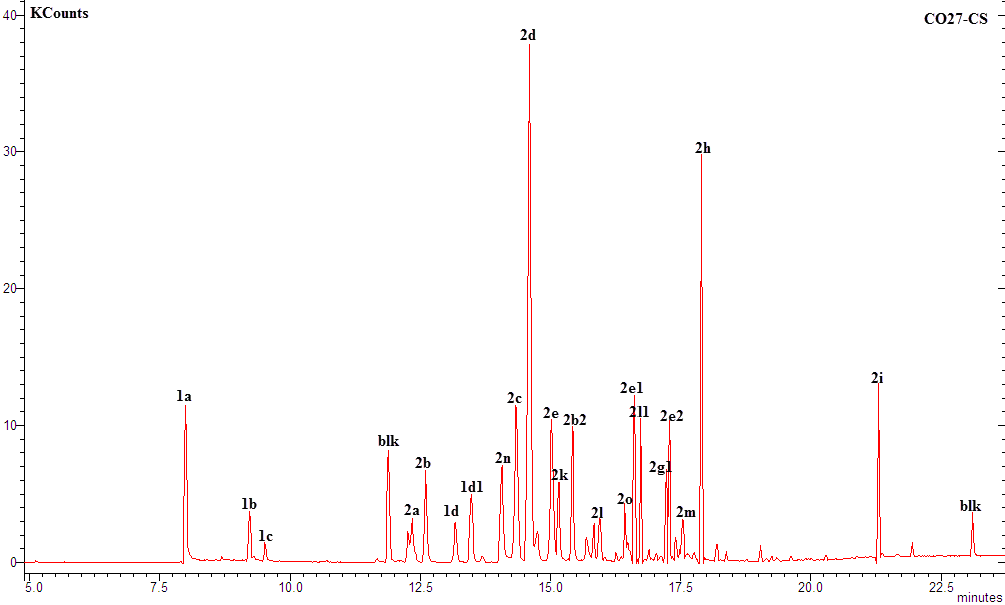


**C**
