# Supplementary material for: Rapid Discovery and Functional Characterization of Terpene Synthases from Four Endophytic Xylariaceae
Source: PLoS One. 2016 Feb 17;11(2):e0146983. doi: 10.1371/journal.pone.0146983 (PMC4757406; doi:10.1371/journal.pone.0146983)
Supplement: S3 Table — (DOCX) [file pone.0146983.s006.docx]

Rapid Discovery and Functional Characterization of Terpene Synthases from Four Endophytic Xylariaceae

Weihua Wu^1^, William Tran^1^, Craig A. Taatjes^2^, Jorge Alonso-Gutierrez^3,4^, Taek Soon Lee^3,4^, John M. Gladden^1,4,^*
^1^ Biomass Science & Conversion Technologies, Sandia National Laboratories, Livermore, CA, USA ^2^Combustion Chemistry Department, Sandia National Laboratories, Livermore, CA, USA; ^3^Physical Biosciences Division, Lawrence Berkeley National Laboratory, Berkeley, CA, USA; ^4^Joint BioEnergy Institute, Emeryville, CA, USA

Supplemental Data

**Table S3.**

| **TPS EC38-CPS from *Hypoxylon sp*. EC38** | | | | |
| --- | --- | --- | --- | --- |
| Compound | Retention Time (min) | % total peak area | Match (%) | R-match (%) |
| **β-chamigrene (3f)** | 17.487 | **34.38** | 88.5 | 88.5 |
| ***β*-pinene (1a)** | 8.003 | **30.71** | 95.2 | 95.5 |
| **limonene(3b)** | 8.699 | **10.23** | 91.5 | 91.9 |
| **2-carene (3a)** | 8.362 | **5.23** | 94.5 | 94.9 |
| *β*-*cis*-Ocimene (**1c**) | 9.518 | 4.0 | 94.6 | 95.4 |
| 4-methyl-3-(1-methylethyldene)-1-cyclohexene (**3d**) | 10.121 | 1.65 | 94.4 | 95.6 |
| β-elemene (**2f**) | 16.475 | 1.02 | 90.7 | 91.1 |
| 1S-*α*-pinene (**1b**) | 9.225 | 1.0 | 90.3 | 96.4 |
| *β* -Linalool (**3e**) | 15.905 | 0.66 | 90.2 | 90.8 |
| **TPS CI4A-CPS from *Hypoxylon sp*. CI4A** | | | | |
| Compound | Retention Time (min) | % total peak area | Match (%) | R-match (%) |
| **β-chamigrene (3f)** | 17.502 | **61.28** | 89 | 89 |
| ***β*-pinene (1a)** | 7.995 | **16.24** | 94.6 | 94.7 |
| limonene(**3b**) | 8.694 | 3.80 | 91.7 | 92.3 |
| *β*-*cis*-Ocimene (**1c**) | 9.517 | 2.53 | 95 | 95.4 |
| 2-carene (**3a**) | 8.355 | 1.46 | 93.8 | 94.6 |
| 1S-*α*-pinene (**1b**) | 9.223 | 0.86 | 88.9 | 95.9 |
| 4-methyl-3-(1-methylethyldene)-1-cyclohexene (**3d**) | 10.116 | 0.854 | 93.5 | 95.1 |
| **TPS EC38-GPS from *Hypoxylon sp*. EC38** | | | | |
| Compound | Retention Time (min) | % total peak area | Match (%) | R-match (%) |
| **α-Gurjunene (2b)** | 17.454 | **20.41** | 92.7 | 94.2 |
| **β-pinene (1a)** | 7.996 | **16.36** | 92.4 | 92.8 |
| **limonene (3b)** | 8.698 | **9.832** | 90.8 | 91.7 |
| β-Elemene (2f) | 16.478 | 4.597 | 91.1 | 92 |
| 2-ethyl-1-hexanol (5b) | 14.753 | 4.321 | 96.4 | 97.2 |
| β-*cis*-Ocimene(1c) | 9.523 | 3.093 | 92.2 | 93.3 |
| L-alloaromadendrene (2l) | 17.142 | 2.44 | 90.4 | 92.1 |
| 4-methyl-3-(1-methylethyldene)-1-cyclohexene (3d) | 10.121 | 2.424 | 94.3 | 95.5 |
| δ-elemene (2f1) | 14.237 | 2.208 | 91.9 | 94.7 |
| β-Farnesene (5c) | 17.023 | 1.697 | 91.8 | 94.5 |
| β-Caryophyllene (2e) | 16.607 | 1.687 | 87.3 | 88.7 |
| (-)-Alloaromadendrene (2l1) | 17.212 | 1.46 | 88.3 | 89.8 |
| (-)-Isoledene (5a) | 16.662 | 1.209 | 92.1 | 93.9 |
| 1S-α-pinene(1b) | 9.225 | 1.078 | 91.7 | 93.1 |
| **TPS CO27-CPS from *Hypoxylon sp*. CO27** | | | | |
| Compound | Retention Time (min) | % total peak area | Match (%) | R-match (%) |
| **β-chamigrene (3f)** | 17.508 | **65.35** | 90.1 | 90.1 |
| ***β*-pinene (1a)** | 7.982 | **10.39** | 93.6 | 93.8 |
| β-elemene (**2f**) | 16.48 | 3.99 | 91.8 | 92 |
| limonene(**3b**) | 8.687 | 3.89 | 91.4 | 91.9 |
| *β*-*cis*-Ocimene (**1c**) | 9.515 | 2.35 | 94.6 | 94.7 |
| (+)-valencene (**2o**) | 17.618 | 1.90 | 95.7 | 97.7 |
| τ-terpinene (**3g**) | 8.346 | 1.50 | 94.5 | 95.3 |
| 4-methyl-3-(1-methylethyldene)-1-cyclohexene (**3d**) | 10.116 | 0.883 | 94.1 | 95.6 |
| 1S-*α*-pinene (**1b**) | 9.22 | 0.59 | 90.8 | 96.4 |


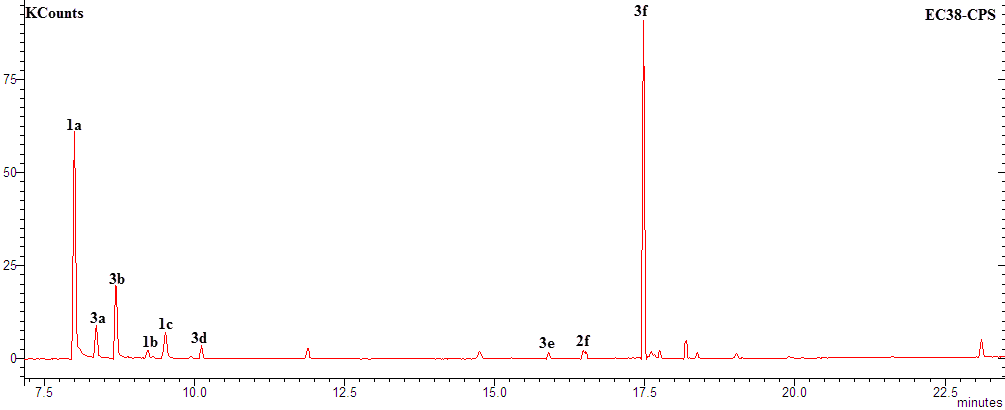


**A**


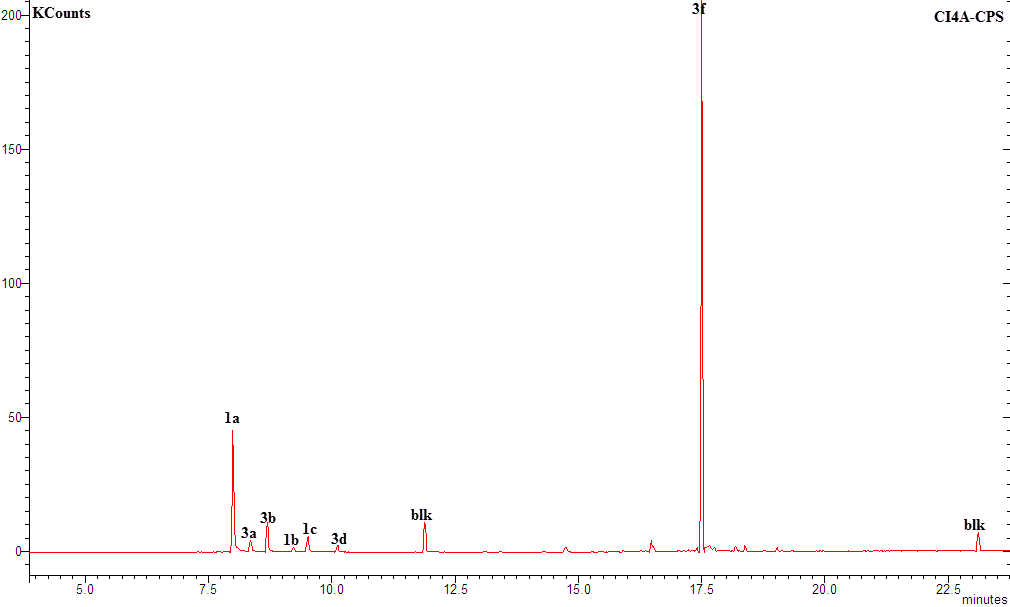


**B**


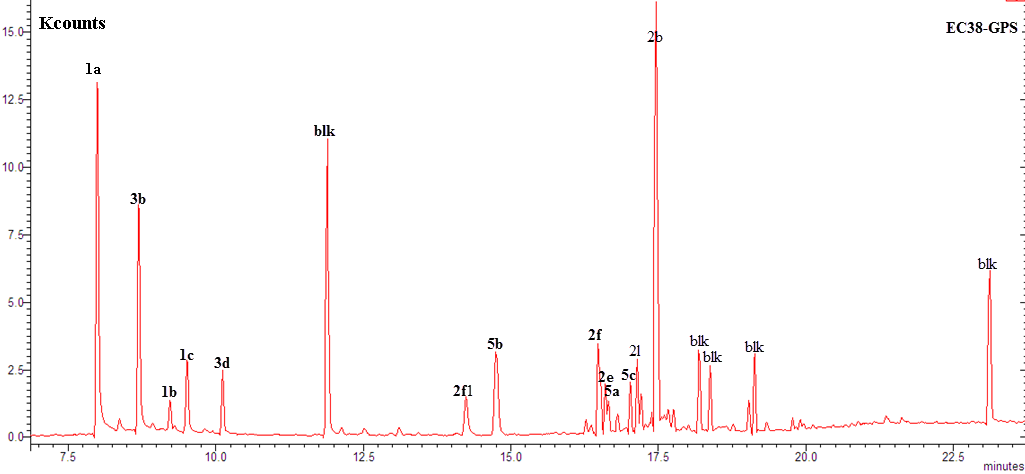


**C**


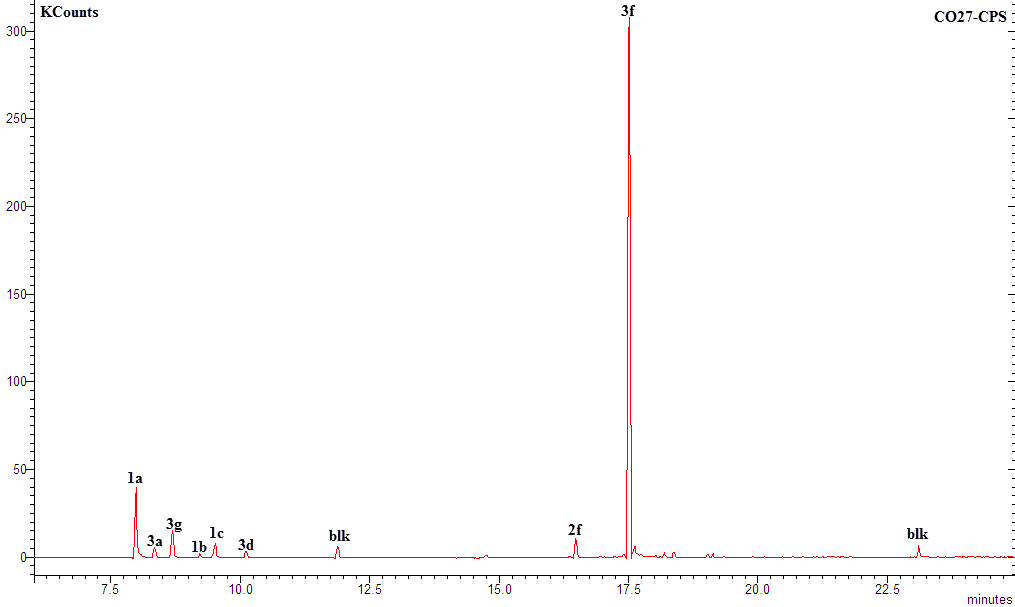


**D**
