# Supplementary material for: Rapid Discovery and Functional Characterization of Terpene Synthases from Four Endophytic Xylariaceae
Source: PLoS One. 2016 Feb 17;11(2):e0146983. doi: 10.1371/journal.pone.0146983 (PMC4757406; doi:10.1371/journal.pone.0146983)
Supplement: S4 Table — (DOCX) [file pone.0146983.s007.docx]

Rapid Discovery and Functional Characterization of Terpene Synthases from Four Endophytic Xylariaceae

Weihua Wu^1^, William Tran^1^, Craig A. Taatjes^2^, Jorge Alonso-Gutierrez^3,4^, Taek Soon Lee^3,4^, John M. Gladden^1,4,^*
^1^ Biomass Science & Conversion Technologies, Sandia National Laboratories, Livermore, CA, USA ^2^Combustion Chemistry Department, Sandia National Laboratories, Livermore, CA, USA; ^3^Physical Biosciences Division, Lawrence Berkeley National Laboratory, Berkeley, CA, USA; ^4^Joint BioEnergy Institute, Emeryville, CA, USA

Supplemental Data

**Table S4.**

| **TPS EC12-GS from *Daldinia eschscholzii* EC12** | | | | |
| --- | --- | --- | --- | --- |
| Compound | Retention Time (min) | % total peak area | Match (%) | R-match (%) |
| **τ-gurjunene (4a1)** | 17.114 | **50.07** | 90.5 | 91.9 |
| **τ-gurjunene (4a)** | 16.484 | **7.96** | 91.7 | 92.8 |
| τ-muurolene (**4b**) | 17.536 | 3.88 | 92 | 94.6 |
| *β*-pinene (**1a**) | 7.994 | 3.71 | 92.6 | 93.8 |
| τ -elemene (**4c**) | 17.748 | 3.44 | 91.6 | 94.4 |
| 1S-*α*-pinene (**1b**) | 9.226 | 3.38 | 93.4 | 95.4 |
| *β*-*cis*-Ocimene (**1c**) | 9.525 | 1.71 | 92.2 | 93.6 |

**
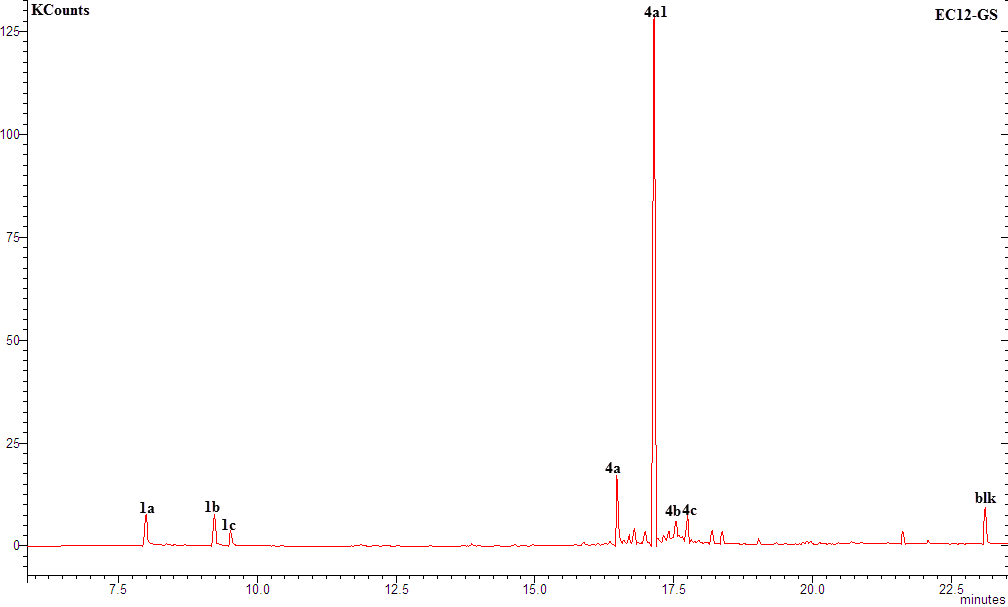
**

**A**
