# Supplementary material for: Rapid Discovery and Functional Characterization of Terpene Synthases from Four Endophytic Xylariaceae
Source: PLoS One. 2016 Feb 17;11(2):e0146983. doi: 10.1371/journal.pone.0146983 (PMC4757406; doi:10.1371/journal.pone.0146983)
Supplement: S5 Table — (DOCX) [file pone.0146983.s008.docx]

Rapid Discovery and Functional Characterization of Terpene Synthases from Four Endophytic Xylariaceae

Weihua Wu^1^, William Tran^1^, Craig A. Taatjes^2^, Jorge Alonso-Gutierrez^3,4^, Taek Soon Lee^3,4^, John M. Gladden^1,4,^*
^1^ Biomass Science & Conversion Technologies, Sandia National Laboratories, Livermore, CA, USA ^2^Combustion Chemistry Department, Sandia National Laboratories, Livermore, CA, USA; ^3^Physical Biosciences Division, Lawrence Berkeley National Laboratory, Berkeley, CA, USA; ^4^Joint BioEnergy Institute, Emeryville, CA, USA

Supplemental Data

**Table S5.**

| **TPS EC12-SS from *Daldinia eschscholzii* EC12** | | | | | |
| --- | --- | --- | --- | --- | --- |
| Compound | | Retention Time (min) | % total peak area | Match (%) | R-match (%) |
| **α-selinene (2h)** | | 17.142 | **50.717** | 91.2 | 93.1 |
| **(-)-Alloaromadendrene (2l)** | | 16.485 | **8.15** | 91.3 | 92.3 |
| **τ -elemene (4c)** | | 17.746 | **6.71** | 93.5 | 96 |
| *β*-pinene (**1a**) | | 7.986 | 4.43 | 94.1 | 94.4 |
| 1S-*α*-pinene (**1b**) | | 9.223 | 4.24 | 94 | 94.8 |
| β-cubebene (**2m**) | | 17.537 | 3.63 | 93.3 | 95.4 |
| *β*-*cis*-Ocimene (**1c**) | | 9.521 | 2.22 | 94 | 94.8 |
| α-gurjunene (**2b**) | | 16.786 | 1.62 | 93.5 | 95.5 |
| **TPS EC12-ILS from *Daldinia eschscholzii* EC12** | | | | | |
| Compound | Retention Time (min) | | % total peak area | Match (%) | R-match (%) |
| **(-)-isoledene(5a)** | 12.652 | | **10.8** | 90.5 | 91 |
| **iso-longifolene (2k)** | 15.622 | | **6.76** | 85.7 | 86.2 |
| **β-caryophyllene (2e)** | 16.605 | | **6.71** | 93.5 | 94.3 |
| β-elemene (**2f**) | 16.485 | | **5.88** | 80.9 | 83.1 |
| (-)-alloaromadendrene (**2l**) | 13.821 | | 2.24 | 85 | 85.7 |
| α-gurjunene (**2b**) | 12.853 | | 1.90 | 85 | 85.9 |
| (+)-valencene (**2o**) | 16.107 | | 1.5 | 86.1 | 88.3 |

**
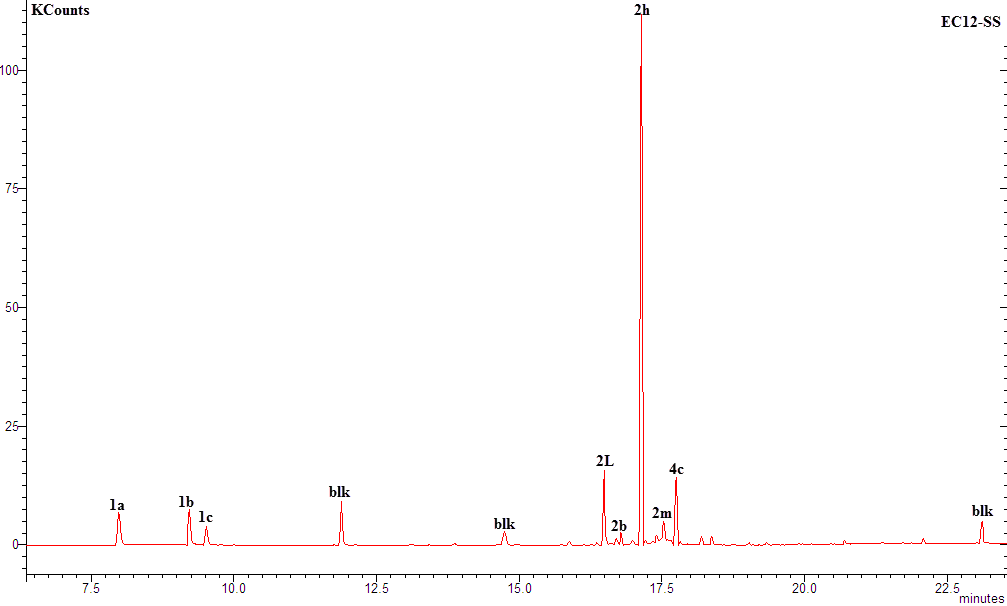
**

**A**


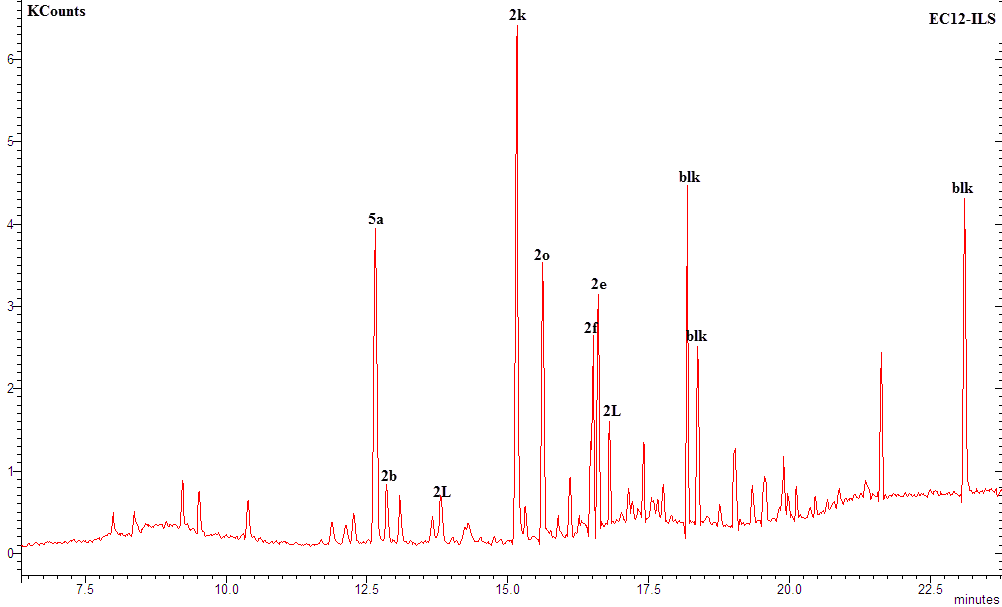


**B**
