# Supplementary material for: Rapid Discovery and Functional Characterization of Terpene Synthases from Four Endophytic Xylariaceae
Source: PLoS One. 2016 Feb 17;11(2):e0146983. doi: 10.1371/journal.pone.0146983 (PMC4757406; doi:10.1371/journal.pone.0146983)

Rapid Discovery and Functional Characterization of Terpene Synthases from Four Endophytic Xylariaceae

Weihua Wu^1^, William Tran^1^, Craig A. Taatjes^2^, Jorge Alonso-Gutierrez^3,4^, Taek Soon Lee^3,4^, John M. Gladden^1,4,^*
^1^ Biomass Science & Conversion Technologies, Sandia National Laboratories, Livermore, CA, USA ^2^Combustion Chemistry Department, Sandia National Laboratories, Livermore, CA, USA; ^3^Physical Biosciences Division, Lawrence Berkeley National Laboratory, Berkeley, CA, USA; ^4^Joint BioEnergy Institute, Emeryville, CA, USA

Supplemental Data

**Table S6.**

| Negative Control (pJBEI3122+ pBbE2k-GPPS_Ag_) | | | | |
| --- | --- | --- | --- | --- |
| Compound | Retention Time (min) | % total peak area | Match (%) | R-match (%) |
| **2-ethyl-hexanolacetate (6a)** | 11.892 | **28.519** | 89 | 92.4 |
| 2-tridecanone (6**d**) | 18.193 | **24.846** | 94 | 94.3 |
| 5H-pyrindine (6h) | 23.114 | **14.198** | 96.2 | 96.5 |
| **ethylhexanol (6b)** | 14.758 | **8.15** | 96.2 | 97.4 |
| **2-undercanone (6c)** | 16.525 | **7.954** | 91.7 | 92.3 |
| 3-eicosene (**6f**) | 19.305 | **6.869** | 94 | 95.4 |
| z-5-decen-1-ol (6e) | 18.38 | **4.951** | 86.2 | 90.6 |
| 2-pentadecanone (**6g**) | 19.908 | 2.423 | 88.2 | 90.7 |


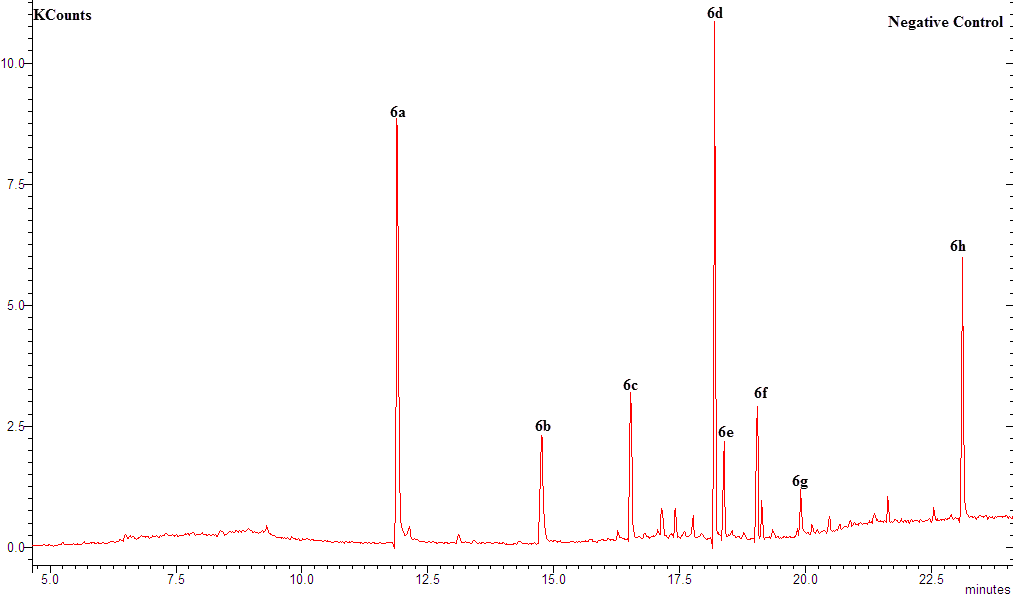

Supplement: S6 Table — (DOCX) [file pone.0146983.s009.docx]
