# Supplementary material for: Rapid Discovery and Functional Characterization of Terpene Synthases from Four Endophytic Xylariaceae
Source: PLoS One. 2016 Feb 17;11(2):e0146983. doi: 10.1371/journal.pone.0146983 (PMC4757406; doi:10.1371/journal.pone.0146983)

Rapid Discovery and Functional Characterization of Terpene Synthases from Four Endophytic Xylariaceae

Weihua Wu^1^, William Tran^1^, Craig A. Taatjes^2^, Jorge Alonso-Gutierrez^3,4^, Taek Soon Lee^3,4^, John M. Gladden^1,4,^*
^1^ Biomass Science & Conversion Technologies, Sandia National Laboratories, Livermore, CA, USA ^2^Combustion Chemistry Department, Sandia National Laboratories, Livermore, CA, USA; ^3^Physical Biosciences Division, Lawrence Berkeley National Laboratory, Berkeley, CA, USA; ^4^Joint BioEnergy Institute, Emeryville, CA, USA

Supplemental Data

**Table S7.**

| Terpene standard | | | | |
| --- | --- | --- | --- | --- |
| Compound | Retention Time (min) | % total peak area | Match (%) | R-match (%) |
| 1R-alpha-pinene | 5.515 | **3.12** | 96.1 | 96.7 |
| Limonene | 8.829 | **12.324** | 89.0 | 92.0 |
| Beta-caryophyllene | 17.454 | **37.631** | 94.2 | 95.4 |
| (+)-valencene | 18.765 | **20.175** | 93.6 | 96.0 |


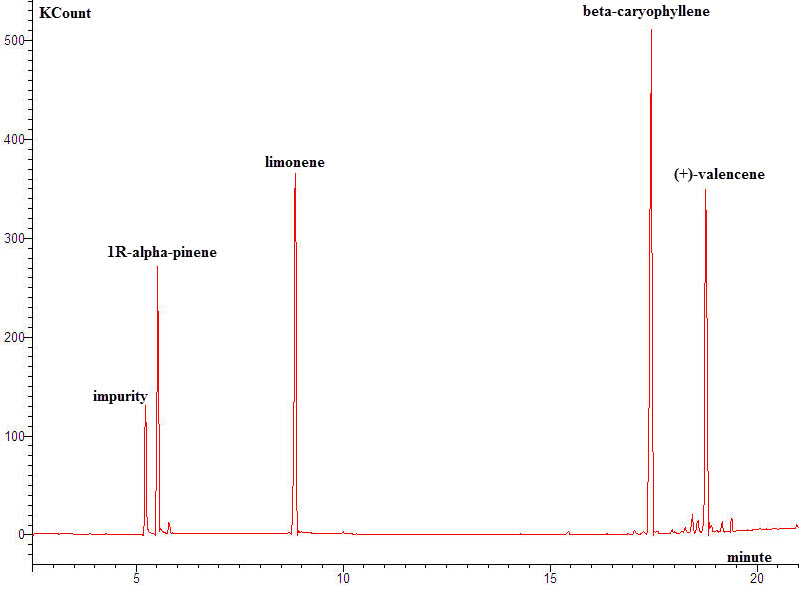

Supplement: S7 Table — (DOCX) [file pone.0146983.s010.docx]
